# Atezolizumab and motixafortide, cobimetinib or simlukafusp alfa in pretreated advanced pancreatic cancer: phase I/IIb MORPHEUS-PDAC umbrella study

**DOI:** 10.1093/oncolo/oyag023

**Published:** 2026-02-25

**Authors:** Gulam A Manji, Vincent Chung, Do-Youn Oh, Jill Lacy, Charles D Lopez, Mariano Ponz-Sarvisé, Teresa Macarulla, Roby Thomas, Ben George, Angela Alistar, Jens T Siveke, Yun Xu, Janet Lau, Edward Cha, Kyu-Pyo Kim, Eileen M O’Reilly

**Affiliations:** Division of Hematology and Oncology, Columbia University Irving Medical Center, New York, NY, 10032, United States; City of Hope National Medical Center, Duarte, CA, 91010, United States; Cancer Research Institute, Seoul National University College of Medicine; Department of Internal Medicine, Seoul National University Hospital; Integrated Major in Innovative Medical Science, Seoul National University Graduate School, Seoul, 03080, Republic of Korea; Department of Medicine, Section of Medical Oncology, Yale School of Medicine, Yale University, New Haven, CT, 06510, United States; Division of Hematology Oncology, Oregon Health & Science University, Knight Cancer Institute, Portland, OR, 97239, United States; Cancer Center Clinica Universidad de Navarra and Program in Solid Tumors (CIMA), Universidad de Navarra, Pamplona, 31008, Spain; Gastrointestinal Cancer Unit, Vall d‘Hebron University Hospital and Vall d‘Hebron Institute of Oncology (VHIO), Barcelona, 08035, Spain; Department of Medical Oncology, University of Pittsburgh Medical Center, Pittsburgh, PA, 15219, United States; Gastrointestinal Oncology, Medical College of Wisconsin, Milwaukee, WI, 53226, United States; Atlantic Hematology Oncology, Morristown, NJ, 07960, United States; Department of Medical Oncology and Division of Solid Tumor Translational Oncology, German Cancer Consortium, West German Cancer Center, University Hospital Essen, Essen, 45147, Germany; Roche (China) Holding Ltd., Shanghai, 201203, China; Genentech, Inc. South San Francisco 94080, United States; Genentech, Inc. South San Francisco 94080, United States; Department of Oncology, Asan Medical Center, University of Ulsan College of Medicine, Seoul, 05505, Republic of Korea; Department of Medicine, Memorial Sloan Kettering Cancer Center, New York, NY, 10065, United States

**Keywords:** pancreatic ductal adenocarcinoma, atezolizumab combination, immunotherapy, CXCR4 inhibitor, MAP/ERK kinase inhibitor, FAP-IL2v

## Abstract

**Background:**

The MORPHEUS platform comprised multiple open-label, randomized, phase Ib/II trials to identify early signals with different treatment combinations across multiple cancers. MORPHEUS-PDAC (NCT03193190) evaluated atezolizumab combinations in pancreatic ductal adenocarcinoma (PDAC). We describe outcomes with atezolizumab plus either motixafortide, cobimetinib, or two simlukafusp alfa regimens.

**Methods:**

Eligible patients with advanced, pretreated PDAC were randomized to receive second-line (2 L) atezolizumab plus either motixafortide (BL8040; *n* = 15), cobimetinib (*n* = 14), simlukafusp alfa every 2 weeks (q2w; *n* = 15), or simlukafusp alfa every 3 weeks (q3w; *n* = 16); or control (mFOLFOX6 [*n* = 25] or gemcitabine plus *nab*-paclitaxel [*n* = 25]). Patients experiencing disease progression or toxicity who met eligibility criteria were enrolled to receive third-line (3 L) atezolizumab plus cobimetinib (*n* = 14), or atezolizumab plus simlukafusp alfa q2w (*n* = 1) or q3w (*n* = 6). Primary endpoints were objective response rates (ORRs) per RECIST 1.1 and safety.

**Results:**

ORRs were 7.1% with atezolizumab-simlukafusp alfa q2w, 8.7% with mFOLFOX6 (both 2 L; 0% in other arms), 14.3% with atezolizumab-cobimetinib, and 16.7% with atezolizumab-simlukafusp alfa q3w (both 3 L). Grade 3-5 adverse event rates were 53.3% (2 L atezolizumab-motixafortide), 64.3% (2 L atezolizumab-cobimetinib), 57.1% (2 L atezolizumab-simlukafusp alfa q2w), 53.3% (2 L atezolizumab-simlukafusp alfa q3w), 63.0% (2 L mFOLFOX6 or gemcitabine-*nab*-paclitaxel), 50.0% (3 L atezolizumab-cobimetinib), and 100% (3 L atezolizumab-simlukafusp alfa q3w).

**Conclusions:**

The overall safety of atezolizumab combinations was manageable and consistent with each agent’s known safety profile. This novel trial design enabled rapid evaluations of 3 atezolizumab combinations; all had limited efficacy as 2 L or 3 L treatment for metastatic PDAC. New treatments are needed to improve outcomes in previously treated PDAC.

Implications for Practice Novel treatments for pancreatic ductal adenocarcinoma (PDAC) are urgently needed, along with faster, more efficient ways to evaluate new molecules or treatment combinations. Although atezolizumab combined with motixafortide, cobimetinib, or simlukafusp alfa had limited efficacy as second- or third-line treatment for metastatic PDAC, the adaptive study design of the randomized phase I/IIb MORPHEUS umbrella trial allowed the simultaneous evaluation of multiple treatment combinations in signal-seeking treatment arms while reducing the number of patients enrolled in shared standard-of-care chemotherapy control arms. This innovative study design could serve as a model for new platform studies in PDAC.

## Introduction

Pancreatic ductal adenocarcinoma (PDAC) is characterized by poor immune cell infiltration and dense fibrotic stroma and is resistant to many antineoplastic treatments, including cancer immunotherapy.^1^ More than 50% of patients present with distant metastases, and their 5-year survival rate is 10%-13%,^2^^,^^3^ highlighting a substantial unmet medical need in this patient population. Despite offering limited survival benefit, chemotherapy combinations have remained the first- and second-line standards of care for patients with metastatic PDAC for more than a decade.^2^^,^^4^^,^^5^

Considering the long clinical development timelines and the number of unsuccessful phase 3 trials in PDAC,^5^ more efficient ways to evaluate new PDAC treatments are necessary, and novel treatments for PDAC are urgently needed. Many trials of cancer immunotherapy in PDAC have involved first-line combinations with chemotherapy, with little success.^6^ However, at the time the MORPHEUS study was designed, it was not known whether novel cancer immunotherapy combinations might benefit patients in whom PDAC had progressed following chemotherapy.

Atezolizumab is an immunotherapy that blocks the programmed cell death ligand 1 (PD-L1), resulting in reduced immunosuppression and restoration of anti-tumor T-cell responses in the tumor microenvironment (TME).^7^ Motixafortide (BL-8040) is a chemokine receptor 4 (CXCR4) inhibitor that can inhibit CXCR4-CXCL12 signaling, promote immune cell infiltration into the TME, thereby turning “cold” tumors “hot,” and increase sensitivity to anti-PD-L1 therapies in pancreatic cancer.^8^^,^^9^ Cobimetinib is a potent and selective MAP/ERK kinase (MEK) inhibitor, a central component of the MAPK pathway.^10–17^ MEK inhibition can increase the expression of MHC-I on tumor cells, enhance T-cell infiltration, and reduce immunosuppression signals in the TME, thereby rendering tumor cells more susceptible to immune attack. Simlukafusp alfa (FAP-IL2v) is an immunocytokine fusion protein that contains a human monoclonal antibody against fibroblast activation protein (FAP) and an engineered variant of interleukin-2 (IL-2v) that preferentially binds the IL-2 receptor on cytotoxic T and NK cells, without binding and activation of regulatory T cells.^18–20^ The FAP antibody domain directs IL-2v into the TME, where FAP is highly expressed on cancer-associated fibroblasts in the pancreatic cancer stroma, and localization of IL-2v in the TME stimulates the proliferation of cytotoxic T and NK cells. In a pancreatic cancer mouse model, combining a murine version of FAP-IL2v with anti-muPD-L1 significantly prolonged survival compared with single-agent treatments.^20^ By combining each of these molecules with atezolizumab, it may be possible to create a more effective and durable immune response to PDAC.

MORPHEUS is an umbrella platform comprising several global, open-label randomized phase Ib/II trials in different tumor types.^21^ MORPHEUS-PDAC allowed the simultaneous evaluation of multiple treatment combinations in signal-seeking treatment arms to rapidly generate evidence of efficacy and safety in PDAC, while limiting enrollment into the standard-of-care chemotherapy control arms.^22^ The adaptive study design also allowed for seamless evaluation and enrollment of patients into multiple treatment lines to evaluate various agents in combination with atezolizumab, an anti-PD-L1 immunotherapy that acts largely by reinvigorating preexisting anti-tumor T-cell responses.^23^^,^^24^

**Figure 1.**
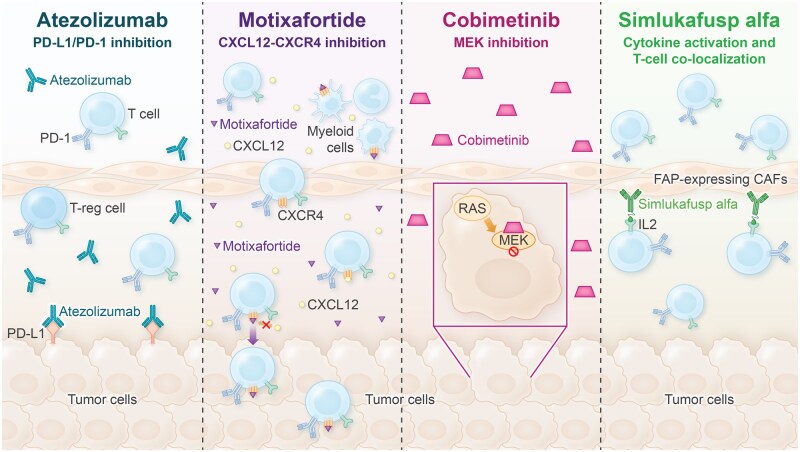
Targeting active pathways in pancreatic cancer. (A) Atezolizumab is a monoclonal antibody that inhibits PD-L1 interactions with PD-1 and B7-1 on T cells, thereby enhancing T-cell responses and improving anti-tumor activity.^23^^,^^24^ In PDAC, PD-L1 expression on tumor cells is a negative prognostic factor. Combining atezolizumab (anti–PD-L1) with other immune modulators in a multi-pronged approach may improve outcomes. (B) Motixafortide is a CXCR4 antagonist that inhibits its interaction with CXCL12 in the tumor microenvironment, which may decrease tumor cell proliferation and migration and increase T-cell infiltration in the tumor microenvironment.^38–44^ (C) Cobimetinib is a MEK inhibitor that blocks tumor cell growth and can increase MHC-I expression as well as tumor antigen presentation and T-cell infiltration.^10–17^ (D) Simlukafusp alfa (FAP-IL2v) is a tumor-targeted interleukin-2 variant (IL2v) fused to a human IgG1 antibody against FAP-ɑ, which is expressed at high levels in PDAC.^18–20^ IL2v preferentially expands and activates CD8+ T cells and natural killer (but not T-regulatory) cells in tumors, an interaction that may be enhanced with anti-PD-L1. Abbreviations: CAF, cancer-associated fibroblasts; CXCL12, cysteine-X-cysteine (C-X-C) motif chemokine ligand 12; CXCR4, C-X-C motif chemokine receptor type 4; FAP, fibroblast activated protein; MEK, mitogen-activated protein kinase; PD-1, programmed cell death protein 1; PD-L1, programmed death ligand 1.

The primary efficacy and safety results for second- and third-line atezolizumab combinations in MORPHEUS-PDAC are reported here. In the second-line setting, atezolizumab was evaluated in combination with either motixafortide, cobimetinib, or simlukafusp alfa. The control treatment was gemcitabine plus *nab*-paclitaxel or mFOLFOX6 chemotherapy. In the third-line setting, atezolizumab was evaluated in combination with either cobimetinib or simlukafusp alfa. Each atezolizumab combination was chosen based on existing preclinical and clinical data and its potential to improve immunotherapy responses either by inhibiting immune suppression, and/or by promoting T-cell priming and activation, trafficking, and/or tumor antigen recognition (Figure 1).

## Methods

### Study design and patients

MORPHEUS-PDAC (NCT03193190) was a global, open-label, two-stage phase Ib/II platform umbrella study. Patients were randomly assigned to one of several experimental arms or to one of two standard-of-care chemotherapy control arms for second-line treatment in Stage 1, as previously described (Figure S1—see online supplementary material for a color version of this figure).^22^ The control arms remained open while experimental arms could be added or removed throughout the study. Patients receiving second-line treatment in Stage 1 who experienced disease progression (PD), unacceptable toxicity, or loss of clinical benefit had the option to enroll into a different third-line treatment combination in Stage 2 if they met the eligibility criteria and an experimental arm was open for enrollment. Randomization into the second-line treatment arms depended on the number of experimental arms that were open at the time of enrollment and arm-specific exclusion criteria, with no more than 35% of patients randomly allocated to control arms at any time.

Eligible patients were aged ≥18 years, had a confirmed diagnosis of metastatic PDAC with measurable disease by Response Evaluation Criteria in Solid Tumors version 1.1 (RECIST 1.1), had PD after treatment with first-line 5-fluorouracil (5-FU)- or gemcitabine-based chemotherapy, and an Eastern Cooperative Oncology Group performance status of 0 or 1. Key exclusion criteria included autoimmune disease; symptomatic, untreated, or actively progressing central nervous system metastases; and active pneumonitis or a history of idiopathic pulmonary fibrosis, organizing pneumonia, or drug-induced or idiopathic pneumonitis.

The study protocol was approved by the institutional review board or ethics committee at each participating center and complied with Good Clinical Practice guidelines, the principles of the Declaration of Helsinki, and local laws. All patients provided written, informed consent.

### Treatment

The treatment regimens in Stage 1 (second line) and Stage 2 (third line) were as follows. In the atezolizumab plus motixafortide arm, patients received a 5-day priming dose of motixafortide: 1.25 mg/kg subcutaneously (SC) on days 1-5. Thereafter, 1200 mg of atezolizumab was administered intravenously (IV) every 3 weeks (q3w) with 1.25 mg/kg of motixafortide SC 3 times a week (days 1, 3, 5, 8, 10, 12, 15, 17, and 19 of each 21-day cycle). In the atezolizumab plus cobimetinib arm, 840 mg of atezolizumab was administered IV every 2 weeks (q2w) with 60 mg of cobimetinib orally on days 1 to 21 of each 28-day cycle. In the atezolizumab plus simlukafusp alfa q2w arm, in the first cycle, 840 mg of atezolizumab was administered IV q2w on days 1 and 15, with 10 mg of simlukafusp alfa administered IV on day 1, and 15 mg of simlukafusp alfa administered IV on days 8, 15, and 22 of a 28-day cycle. All subsequent cycles were followed with 840 mg of atezolizumab IV q2w and 15 mg of simlukafusp alfa IV q2w of each 28-day cycle. In the atezolizumab plus simlukafusp alfa q3w arm, 1200 mg of atezolizumab was administered IV q3w with 10 mg of simlukafusp alfa IV q3w of each 21-day cycle.

In the control arm, patients were administered either gemcitabine plus *nab*-paclitaxel (125 mg/m^2^  *nab*-paclitaxel with 1000 mg/m^2^ gemcitabine, both IV on days 1, 8, and 15 of each 28-day cycle) or mFOLFOX6 chemotherapy (85 mg/m^2^ oxaliplatin, 400 mg/m^2^ leucovorin and 400 mg/m^2^ bolus 5-FU, all IV on days 1 and 15 of each 28-day cycle, with 2400 mg/m^2^ of continuous 5-FU IV on days 1, 2, 15, and 16 of each 28-day cycle).

Treatment continued until the patient experienced unacceptable toxicity and/or loss of clinical benefit, as determined by the investigator, or PD per RECIST 1.1. Tumor assessments were done at baseline, every 6 weeks for the first 48 weeks, and every 12 weeks thereafter until radiographic PD per RECIST 1.1. Patients in the experimental arms who continued treatment after radiographic PD underwent tumor assessments every 6 weeks until loss of clinical benefit.

### Endpoints

The primary endpoint was the investigator-assessed objective response rate (ORR) per RECIST 1.1, defined as the proportion of patients with a complete response (CR) or partial response (PR) on 2 consecutive occasions >4 weeks apart. Key secondary endpoints included investigator-assessed progression-free survival (PFS), disease control rate (DCR), and duration of response (DOR), all per RECIST 1.1, and overall survival (OS). The primary safety endpoint was the incidence of participants with adverse events (AEs) with severity determined according to the National Cancer Institute Common Terminology Criteria for Adverse Events, version 4.0.

### Statistical analyses

**Figure 2.**
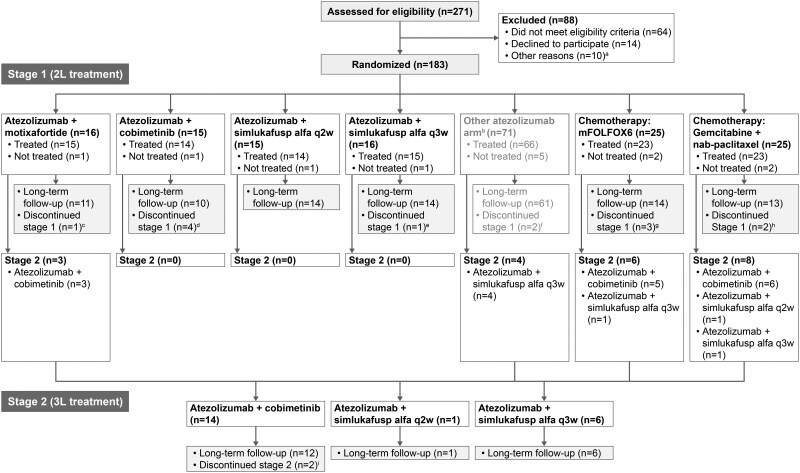
Trial profile. ^a^Investigator withdrawal of patient (*n* = 6); no reason provided (*n* = 3); death (*n* = 1); ^b^Atezolizumab + PEGPH20 (data presented elsewhere (8)); ^c^Withdrawal by subject (*n* = 1); ^d^Death (*n* = 1); withdrawal by subject (*n* = 3); ^e^Other (*n* = 1); ^f^Death (*n* = 1); physician decision (*n* = 1); ^g^Death (*n* = 3); withdrawal by subject (*n* = 1); other (*n* = 3) ^h^Death (*n* = 1); withdrawal by subject (*n* = 4); other (*n* = 3); ^i^Death (*n* = 1). Abbreviations: 2L, second line; 3L, third line; mFOLFOX6, 5-fluorouracil, leucovorin, and oxaliplatin; q2w, every 2 weeks; q3w, every 3 weeks.

This study was designed to obtain preliminary efficacy and safety data on atezolizumab treatment combinations in patients with PDAC, not to make explicit power and type I error considerations for a hypothesis test, as previously described.^22^^,^^25^ Approximately 15 patients were enrolled into each treatment arm during the Stage 1 preliminary phase (Figure 2). If a clinically meaningful improvement in ORR was observed in the treatment arm relative to the control arms, with added consideration of the totality of efficacy (PFS and OS) and safety data available, indicating clinical activity in the absence of unacceptable toxicity, an additional 25 patients for that arm could be enrolled into the expansion phase. Efficacy was evaluated in all patients who received at least one dose of each drug; safety was evaluated in all patients who received any amount of study treatment. The ORR was calculated for each treatment arm, along with 95% confidence intervals (CIs) using the Clopper-Pearson method. Patients with missing or no response assessments were classified as non-responders. Median DOR (in efficacy-evaluable patients who had a confirmed CR or PR), PFS, and OS were estimated using the Kaplan-Meier method, with 95% CIs constructed using the Brookmeyer and Crowley method. For patients who did not have documented PD or death, PFS and DOR were censored at the last tumor assessment. Patients alive at the OS analysis cutoff were censored at the last date they were known to be alive.

## Results

### Patient disposition and baseline characteristics

Between July 5, 2017, and September 10, 2019, 183 eligible patients at 18 sites were randomized into MORPHEUS-PDAC (Figure 2; Table S1—see online supplementary material), of which 104 patients received at least one dose of second-line treatment in Stage 1 with either atezolizumab plus motixafortide (*n* = 15), atezolizumab plus cobimetinib (*n* = 14), atezolizumab plus simlukafusp alfa q2w (*n* = 14), atezolizumab plus simlukafusp alfa q3w (*n* = 15), mFOLFOX6 (*n* = 23), or gemcitabine plus *nab*-paclitaxel (*n* = 23). An additional 66 patients received a different atezolizumab-based second-line treatment (atezolizumab plus PEGPH20) described elsewhere.^22^

Overall, 21 eligible patients (3 patients from the second-line atezolizumab plus cobimetinib arm, 4 from the second-line atezolizumab plus PEGPH20 arm,^22^ and 14 from the second-line control arms) entered Stage 2 and received at least 1 dose of third-line treatment with either atezolizumab plus cobimetinib (*n* = 14), atezolizumab plus simlukafusp alfa q2w (*n* = 1), or atezolizumab plus simlukafusp alfa q3w (*n* = 6) (Figure 2; Table S1—see online supplementary material). Nineteen patients (90%) had long-term follow-up. The most frequent reason for treatment discontinuation were PD (50%-100% of patients in Stage 1 and 36%-100% in Stage 2), followed by patient withdrawal (0%-29% in Stage 1 and 0%-14% in Stage 2 arms). Death was the most frequent reason for study discontinuation (67%-100% in each treatment arm) (Table S1—see online supplementary material). The clinical cutoff date was October 14, 2019, for all arms except for the atezolizumab plus simlukafusp alfa arms, for which the clinical cutoff date was June 25, 2021.

Baseline characteristics of the patients are summarized by treatment arm in Table 1 and Table S2 (see online supplementary material). The majority of patients were Asian or White. In each treatment arm, the median age ranged from 58.5 to 73 years, the median number of metastatic sites was 1 to 2.5, and ≥66.7% of the patients had liver metastases.

**Table 1.** Baseline characteristics.

	Stage 1 (second-line treatment)						Stage 2 (third-line treatment)		
	Atezo + moti (*n* = 15)	Atezo + cobi (*n* = 14)	Atezo + sim q2w (*n* = 15)	Atezo + sim q3w (*n* = 16)	**mFOLFOX6** ** (*n* = 25)**	Gem + *nab*-P (*n* = 25)	Atezo + cobi (*n* = 14)	Atezo+ sim q2w (*n* = 1)	Atezo + sim q3w (*n* = 6)
**Age, years**									
** Median (range)**	67 (44-77)	59.5 (46-77)	63 (43-83)	64.5 (39-82)	67 (53-78)	58 (39-73)	66.5 (46-77)	73 (73-73)	58.5 (37-83)
** ≥65, *n* (%)**	8 (53.3)	5 (35.7)	7 (46.7)	8 (50.0)	16 (64.0)	5 (20.0)	8 (57.1)	1 (100)	1 (16.7)
**Male, *n* (%)**	10 (66.7)	9 (64.3)	8 (53.3)	7 (43.8)	11 (44.0)	14 (56)	6 (42.9)	1 (100)	3 (50.0)
**Race, *n* (%)**									
** Asian**	8 (53.3)	4 (28.6)	3 (20.0)	6 (37.5)	11 (44.0)	6 (24.0)	2 (14.3)	0	3 (50.0)
** White**	6 (40.0)	8 (57.1)	11 (73.3)	10 (62.5)	14 (56.0)	19 (76.0)	12 (85.7)	1 (100)	3 (50.0)
** Other**	1 (6.7)	2 (14.3)	1 (6.7)	0	0	0	0	0	0
**ECOG PS, *n* (%)**									
** 0**	8 (53.3)	4 (28.6)	3 (21.4)	7 (43.8)	8 (32.0)	11 (44.0)	3 (21.4)	1 (100)	1 (16.7)
** 1**	7 (46.7)	10 (71.4)	11 (78.6)	9 (56.3)	17 (68.0)	14 (56.0)	10 (71.4)	0	5 (83.3)
** 2**	0	0	0	0	0	0	1 (7.1)	0	0
**Metastatic sites**									
** Median**	2.0	3.0	1.0	1.0	2.0	2.0	2.5	2.0	1.5
** Range**	1-5	1-5	1-2	1-3	1-6	1-3	2-6	2-2	1-3
** Liver metastases, *n* (%)**	10 (66.7)	11 (78.6)	11 (73.3)	11 (68.8)	19 (76.0)	17 (68.0)	11 (78.6)	1 (100)	5 (83.3)
**Prior chemotherapy, *n* (%)**									
** 5-FU**	9 (60.0)	8 (57.1)	5 (33.3)	7 (43.8)	0	25 (50.0)	7 (50.0)	1 (100)	4 (66.7)
** Gemcitabine**	6 (40.0)	6 (42.9)	10 (66.7)	9 (56.3)	25 (50.0)	0	7 (50.0)	0	2 (33.3)
**Prior cancer surgery, *n* (%)**	1 (6.7)	3 (21.4)	4 (26.7)	3 (18.8)	3 (12.0)	5 (20.0)	3 (21.4)	0	1 (16.7)
**Prior radiotherapy, *n* (%)**	1 (6.7)	3 (21.4)	2 (13.3)	3 (18.8)	0	5 (20.0)	2 (14.3)	0	1 (16.7)

Abbreviations: Atezo, atezolizumab; cobi, cobimetinib; ECOG PS, Eastern Cooperative Oncology Group performance status; FU, fluorouracil; gem, gemcitabine; mFOLFOX-6, 5-fluorouracil, leucovorin, and oxaliplatin; *nab*-P, *nab*-paclitaxel; q2w, every 2 weeks; q3w, every 3 weeks; sim, simlukafusp alfa.

### Efficacy of second-line treatment in Stage 1

**Figure 3.**
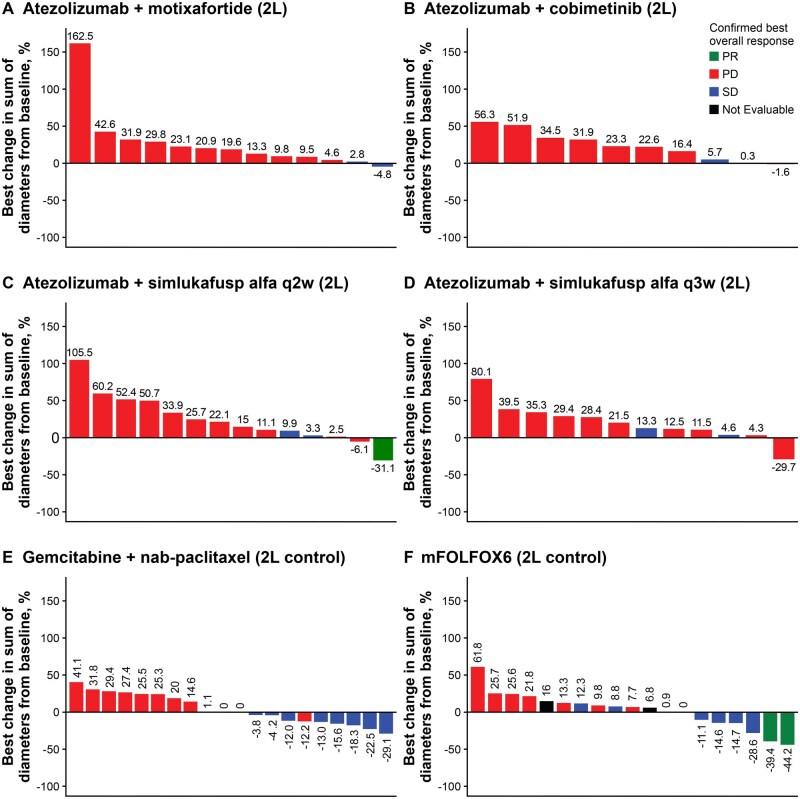
Clinical activity with second-line treatment combinations in Stage 1. Abbreviations: 2L, second line; mFOLFOX6, 5-fluorouracil, leucovorin, and oxaliplatin; PD, disease progression; PR, partial response; q2w, every 2 weeks; q3w, every 3 weeks; SD, stable disease.

In Stage 1, the median duration of survival follow-up ranged from 4.07 to 7.33 months (Table 2). The best overall response with these second-line treatment combinations was PR. PRs were observed in 1/14 patients treated with atezolizumab plus simlukafusp alfa (q2w) (ORR 7.1% [95% CI, 0.18, 33.87]), and 2/23 patients treated with mFOLFOX6 in the control arm (ORR 8.7% [95% CI, 1.07, 28.04]) (Table 2, Figure 3, Figure S2—see online supplementary material). Exploratory biomarker analysis showed that the patient with a PR to atezolizumab plus simlukafusp alfa (q2w) had microsatellite instability-high (MSI-H) status and an inflamed immune phenotype.^26^ No PRs or CRs were seen in any other experimental arm or in the control gemcitabine plus *nab*-paclitaxel arm. The DCRs in Stage 1 mirrored the ORRs of 0% to 7.1% observed with the second-line atezolizumab combinations; DCRs with control treatment were 30.4% (7/23 patients; 95% CI, 13.21, 52.92) in the mFOLXOX6 arm and 34.8% (8/23 patients; 95% CI, 16.38, 57.27) in the gemcitabine plus *nab*-paclitaxel arm. Among patients who had a response, the median DOR was 6.14 months (95% CI, not estimable [NE]) in the atezolizumab plus simlukafusp alfa q2w arm and 3.37 months (95% CI, 2.83, NE) in the mFOLFOX6 arm.

**Table 2.** Clinical activity in MORPHEUS-PDAC.

	Stage 1 (second-line treatment)						Stage 2 (third-line treatment)		
	Atezo + moti (*n* = 15)	Atezo + cobi (*n* = 14)	Atezo + sim q2w (*n* = 14)	Atezo + sim q3w (*n* = 15)	mFOLFOX6 (*n* = 23)	Gem + *nab*-P (*n* = 23)	Atezo + cobi (*n* = 14)	Atezo + sim q2w (*n* = 1)	Atezo + sim q3w (*n* = 6)
**Median duration of survival follow-up, months**	4.53	4.07	7.33	4.4	6.64	7.03	5.95	0.85	4.39
**ORR, *n* (%)** **[95% CI]**	0 [0.00, 23.16]	0 [0.00, 23.16]	1 (7.1) [0.18, 33.87]	0 [0.00, 21.80]	2 (8.7) [1.07, 28.04]	0 [0.00, 14.82]	2 (14.3) [1.78, 42.81]	0 [0.00, 97.50]	1 (16.7) [0.42, 64.12]
** CR**	0 [0.00, 23.16]	0 [0.00, 23.16]	0 [0.00, 23.16]	0 [0.00, 21.80]	0 [0.00,14.82]	0 [0.00, 14.82]	0 [0.00, 23.16]	0 [0.00, 97.50]	0 [0.00, 45.93]
** PR**	0 [0.00, 23.16]	0 [0.00, 23.16]	1 (7.1) [0.18, 33.87]	0 [0.00, 21.80]	2 (8.7) [1.07, 28.04]	0 [0.00, 14.82]	2 (14.3) [1.78, 42.81]	0 [0.00, 97.50]	1 (16.7) [0.42, 64.12]
**SD**	2 (14.3) [1.78, 42.81]	2 (14.3) [1.78, 42.81]	2 (14.3) [1.78, 42.81]	2 (13.3) [1.66, 40.46]	7 (30.4) [13.21, 52.92]	11 (47.8) [26.82, 69.41]	3 (21.4) [4.66, 50.80]	0 [0.00, 97.50]	1 (16.7) [0.42, 64.12]
**PD**	11 (78.6) [49.20, 95.34]	8 (57.1) [28.86, 82.34]	11 (78.6) [49.20, 95.34]	10 (66.7) [38.38, 88.18]	8 (34.8) [16.38, 57.27]	9 (39.1) [19.71, 61.46]	4 (28.6) [8.39, 58.10]	1 (100) [2.50, 100.00]	2 (33.3) [4.33, 77.72]
**NE**	0	0	0	1 (6.7)	2 (8.7)	0	1 (7.1)	0	0
**Missing**	1 (7.1)	4 (28.6)	0	2 (13.3)	4 (17.4)	3 (13.0)	4 (28.6)	0	2 (33.3)
**Disease control rate, *n* (%) [95% CI]**	0 [0.00, 23.16]	0 [0.00, 23.16]	1 (7.1) [0.18, 33.87]	0 [0.00, 21.80]	7 (30.4) [13.21, 52.92]	8 (34.8) [16.38, 57.27]	5 (35.7) [12.76, 64.86]	0 [0.00, 97.50]	2 (33.3) [4.33, 77.72]
**Median DOR (95% CI)**	–	–	6.14 (NE)	–	3.37 (2.83, NE)	–	4.9 (4.07, 5.72)	–	5.55 (NE)
**PFS**									
** Patients with event, *n* (%)**	14 (100)	11 (78.6)	14 (100)	15 (100)	20 (87.0)	22 (95.7)	14 (100)	1 (100)	6 (100)
** Median, months ** **(95% CI)**	1.64 (1.41, 1.87)	1.28 (1.15, 1.84)	1.46 (1.31, 1.58)	1.38 (1.38, 2.66)	2.76 (1.45, 5.52)	2.53 (1.77, 4.04)	2.22 (1.38, 5.45)	0.26 (NE)	1.72 (1.41, 4.67)
**OS**									
** Patients with event, *n* (%)**	14 (100)	11 (78.6)	13 (92.9)	14 (93.3)	19 (82.6)	21 (91.3)	12 (85.7)	1 (100)	5 (83.3)
** Median, months (95% CI)**	5.19 (3.25, 8.87)	5.49, (2.69. 6.97)	7.33 (4.86, 11.60)	4.7 (3.81, 11.04)	6.8 (6.47, 9.66)	7.03 (4.11, 10.68)	6.60 (1.87, 10.45)	0.85 (NE)	6.83 (1.94, NE)

Abbreviations: Atezo, atezolizumab; cobi, cobimetinib; CR, complete response; DOR, duration of response; gem, gemcitabine; mFOLFOX-6, 5-fluorouracil, leucovorin, and oxaliplatin; *nab*-P, *nab*-paclitaxel; NE, not evaluable; ORR, objective response rate; OS, overall survival; PD, disease progression; PFS, progression-free survival; PR, partial response; q2w, every 2 weeks; q3w, every 3 weeks; SD, stable disease; sim, simlukafusp alfa.

The median PFS ranged from 1.28 (95% CI, 1.15, 1.84) months in the atezolizumab plus cobimetinib arm (*n* = 14) to 1.64 (95% CI, 1.41, 1.87) months in the atezolizumab plus motixafortide arm (*n* = 15; Table 2; Figure S4—see online supplementary material). In the control arm, the median PFS was 2.76 (95% CI, 1.45, 5.52) months in the mFOLFOX6 arm (*n* = 23) and 2.53 (95% CI, 1.77, 4.04) months in the gemcitabine plus *nab*-paclitaxel arm (*n* = 23).

Among atezolizumab combination therapies, the median OS was longest with second-line atezolizumab plus simlukafusp alfa q2w (*n* = 14; 7.33 [95% CI, 4.86, 11.60] months); this was similar to chemotherapy in second-line with mFOLFOX6 (6.8 [95% CI, 6.47, 9.66] months) and gemcitabine plus *nab*-paclitaxel (7.03 [95% CI, 4.11, 10.68] months) (Table 2; Figure S4—see online supplementary material). The median OS was approximately 5 months with the other second-line atezolizumab combinations. All experimental arms were closed at the end of the preliminary phase due to the limited responses observed.

### Efficacy of third-line treatment in Stage 2

In Stage 2, the median duration of survival follow-up was 5.95 months in the atezolizumab plus cobimetinib arm, 0.85 months in the atezolizumab plus simlukafusp alfa q2w arm, and 4.39 months in the atezolizumab plus simlukafusp alfa q3w arm (Table 2). Two of 14 patients in the third-line atezolizumab plus cobimetinib arm had a PR (ORR 14.3% [95% CI, 1.78, 42.81]; Figure S5—see online supplementary material); both had received chemotherapy as their second-line treatment without any response. Based on limited exploratory biomarker analyses, one patient had microsatellite stable (MSS) PDAC with an immune desert phenotype, and the other patient’s MSI status is unknown, but the tumor immune phenotype was inflamed.^27^ In the third-line atezolizumab plus simlukafusp alfa q3w arm, 1/6 patients had a PR (ORR 16.7% [95% CI, 0.42, 64.12]); this patient had received a second-line atezolizumab-based therapy, had MSS PDAC and an inflamed immune phenotype.^26^ Lastly, one patient who had received second-line chemotherapy transitioned to receive third-line atezolizumab plus simlukafusp alfa q2w. After receiving the initial doses of treatment, they demonstrated radiographic disease progression 7 days later with no response to treatment.

The DCRs were 35.7% (4/14; 95% CI, 12.76, 64.86) with atezolizumab plus cobimetinib and 33.3% (2/6; 95% CI, 4.33, 77.72) with atezolizumab plus simlukafusp alfa q3w. The median DOR was 4.9 months (95% CI, 4.07, 5.72) in the atezolizumab plus cobimetinib arm and 5.55 months (95% CI was not evaluable) in the atezolizumab plus simlukafusp alfa q3w arm.

The median PFS was 2.22 months (95% CI, 1.38, 5.45) in the atezolizumab plus cobimetinib arm (*n* = 14), 0.26 months (95% CI, NE) in the atezolizumab plus simlukafusp alfa q2w arm (*n* = 1), and 1.72 months (95% CI, 1.41, 4.67) in the atezolizumab plus simlukafusp alfa q3w arm (*n* = 6; Table 2; Figure S5A—see online supplementary material).The median OS after third-line atezolizumab plus cobimetinib, atezolizumab plus simlukafusp alfa q2w, and atezolizumab plus simlukafusp alfa q3w were 6.60 months (*n* = 14; 95% CI, 1.87, 10.45), 0.85 months (*n* = 1; 95% CI, NE), and 6.83 months (*n* = 6; 95% CI, 1.94, NE), respectively (Table 2; Figure S6B—see online supplementary material).

### Safety

The median duration of exposure to each treatment combination, along with the safety summary, is presented in Table 3. All patients treated with an atezolizumab combination in Stages 1 and 2 reported at least one AE, except the single patient who received third-line atezolizumab plus simlukafusp alfa q2w in Stage 2. The majority of AEs in each arm were grade 1-3. Grade 5 AEs occurred in one patient treated with second-line chemotherapy (2.2%; disseminated intravascular coagulation related to mFOLFOX6 ­treatment) and one patient in the third-line atezolizumab plus cobimetinib arm (7.1%; systemic candida unrelated to treatment).

**Table 3.** Safety summary.

	Stage 1 (second-line treatment)					Stage 2 (third-line treatment)		
	Atezo + moti (*n* = 15)	Atezo + cobi (*n* = 14)	Atezo + sim q2w (*n* = 14)	Atezo + sim q3w (*n* = 15)	Gem + *nab*-P or mFOLFOX6 (*n* = 46)	Atezo + cobi (*n* = 14)	Atezo + sim q2w (*n* = 1)	Atezo + sim q3w (*n* = 6)
**Duration of treatment, median (range), days**	Atezo: 22 (1-64) Moti: 45 (5-89)	Atezo: 17.5 (1-78) Cobi: 21.5 (7-85)	Atezo: 30 (15-354) Sim: 30 (16-354)	Atezo: 23 (1-66) Sim: 23 (1-66)	*nab*-P: 49 (1-297) Gem: 71 (1-297) Oxa: 33 (1-256) 5-FU bolus: 29 (1-256) 5-FU continuous: 35 (2-276) Leucovorin: 33 (1-256)	Atezo: 39.5 (1-351) Cobi: 49 (1-365)	Atezo: 1 Sim: 1	Atezo: 22.5 (22-319) Sim: 22.5 (22-319)
**TRAE, *n* (%)**	15 (100)	11 (78.6)	13 (92.9)	13 (86.7)	40 (87.0)	9 (64.3)	0	5 (83.3)
**TR SAE, *n* (%)**	1 (6.7)	2 (14.3)	0	5 (33.3)	8 (17.4)	2 (14.3)	0	0
**TRAE leading to withdrawal from treatment, *n* (%)**	0	0	0	0	1 (2.2)	1 (7.1)	0	0
**TRAE leading to dose modification/interruption, *n* (%)**	3 (20.0)	5 (35.7)	3 (21.4)	1 (6.7)	29 (63.0)	4 (28.6)	0	0
**AESI, *n* (%)^a^**	6 (40)	10 (71.4)	8 (57.1)	9 (60)	18 (39.1)	2 (14.3)	0	4 (66.7)

Abbreviations: AE, adverse event; AESI, adverse event of special interest; Atezo: atezolizumab; cobi, cobimetinib; gem, gemcitabine; mFOLFOX-6, 5-fluorouracil, leucovorin, and oxaliplatin; *nab*-P, *nab*-paclitaxel; q2w, every 2 weeks; q3w, every 3 weeks; SAE, serious adverse event; sim, simlukafusp alfa.

a Sponsor-defined.

Serious adverse events (SAEs) with second-line treatment occurred in 4 patients (26.7%) with atezolizumab plus motixafortide, 8 patients (57.1%) with atezolizumab plus cobimetinib, 2 patients (14.3%) with atezolizumab plus simlukafusp alfa q2w, 7 patients (46.7%) with atezolizumab plus simlukafusp alfa q3w, and 22 patients (47.8%) with chemotherapy. SAEs with third-line treatment occurred in 7 patients (50%) with atezolizumab plus cobimetinib and 2 patients (33%) with atezolizumab plus simlukafusp alfa q3w.

Adverse events of special interest (AESIs) with second-line treatment occurred in 6 patients (40%) with atezolizumab plus motixafortide, 10 patients (71.4%) with atezolizumab plus cobimetinib, 8 patients (57.1%) with atezolizumab plus simlukafusp alfa q2w, 9 patients (60%) with atezolizumab plus simlukafusp alfa q3w, and 18 patients (39.1%) with chemotherapy (Table 3). AESIs with third-line treatment occurred in 2 patients (14.3%) with atezolizumab plus cobimetinib and 4 patients (66.7%) with atezolizumab plus simlukafusp alfa q3w.

Treatment-related AEs (TRAEs) occurred in 64.3% to 100% of patients across treatment arms and settings (Table 3) except the third-line atezolizumab plus simlukafusp alfa q2w arm (*n* = 1, no AEs). The most common TRAEs with each atezolizumab combination were fatigue with atezolizumab plus motixafortide in 8 patients (53.3%), diarrhea with atezolizumab plus cobimetinib (in 5 patients [35.7%] as second-line treatment and 4 patients [42.9%] as third-line treatment), nausea with atezolizumab plus simlukafusp alfa q2w (in 8 patients [57.1%] in the q2w arm as second-line treatment), and pyrexia with atezolizumab plus simlukafusp alfa q3w (in 8 patients [53.3.1%] as second-line treatment and 3 patients [50.0%] as third-line treatment and 2 patients [33.3%]) in the q3w arm as third-line treatment (Table S3—see online supplementary material). Nausea was the most common TRAE in the chemotherapy arm (18 patients [39.1%]).

## Discussion

This large, global, randomized, umbrella-platform study enabled the simultaneous evaluation of the efficacy and safety of several experimental combinations of atezolizumab with motixafortide, cobimetinib or simlukafusp alfa as second- or third-line treatment for patients with metastatic PDAC that had progressed following prior chemotherapy. This study design was adapted from the previously described “rolling arms” trial that allowed several experimental treatments to be evaluated with smaller sample size requirements, fewer patients treated with standard of care, and shorter time to novel treatment discovery.^28^ Although limited clinical activity was observed with these atezolizumab combinations, the novel, adaptive, and dynamic signal-seeking design of the MORPHEUS platform allowed multiple treatment combinations to be evaluated quickly (by using an ORR primary endpoint), efficiently (using a shared comparator control and multiple experimental treatment options across different treatment settings), and robustly (by using a global, randomized, controlled design), in contrast to typical single-arm phase I/II trials. The shared randomized control arm was a study strength that not only permitted efficient use of the study population by minimizing the number of patients allocated to standard-of-care chemotherapy but also increased confidence in data interpretation. Additionally, this study design gave patients opportunities to access multiple treatment options across 2 treatment settings using a gated enrollment, which prevented enrollment to treatments with limited benefit. The crossover study design from Stage 1 to 2 allowed longitudinal monitoring of tumor activity while allowing patients to move seamlessly from one therapy and treatment line to another within a single study. Finally, the MORPHEUS platform was effective in forging collaborations among pharmaceutical partners in the search for new treatments for metastatic PDAC.

At present, gemcitabine-based and fluoropyrimidine-based chemotherapy are recommended to alternate as first- and second-line standard-of-care therapies for pancreatic cancer in the European Society for Medical Oncology and National Comprehensive Cancer Network (NCCN) guidelines; ^29^^,^^30^ however, no randomized clinical trial has ever directly compared these regimens in the second-line setting. Here, MORPHEUS-PDAC provided the first prospectively randomized efficacy and safety data comparing second-line gemcitabine plus *nab*-paclitaxel and mFOLFOX6. Although no CRs or PRs were seen in the gemcitabine plus *nab*-paclitaxel arm and only one PR was observed in the mFOLFOX6 arm, second-line gemcitabine plus *nab*-paclitaxel and mFOLFOX6, respectively, resulted in DCRs of 34.8% and 30.4%, median PFS of 2.53 and 2.76 months, and median OS of 7.75 and 6.80 months. These chemotherapy regimens were generally tolerable, with one treatment-related death (2.2%) and only 4.3% of patients discontinuing treatment due to an AE. These efficacy outcomes compared favorably with survival outcomes obtained with second-line 5-FU and gemcitabine-based therapy (as single agents or in combinations) in a large Chinese real-world study.^31^ This small randomized dataset from MORPHEUS-PDAC demonstrated no measurable difference in outcomes, indicating that patients can benefit either from gemcitabine plus *nab*-paclitaxel or from mFOLFOX6 as second-line treatment. Moreover, these results appear to be equivalent to the only approved second-line regimen of liposomal irinotecan in combination with 5-FU and leucovorin, which demonstrated a median PFS of 3.1 months and median OS of 6.2 months in the NAPOLI-1 trial.^32^

Overall, the atezolizumab combinations evaluated in MORPHEUS-PDAC were tolerable, with treatment-related toxicities being consistent with the known AE profiles of the individual drugs.^12^^,^^13^^,^^33^^,^^34^ Findings from MORPHEUS-PDAC highlight that, despite some signals of clinical activity, drug development for later-line treatment of metastatic PDAC remains challenging. The second-line results suggest that inhibiting the PD-L1 pathway along with MEK (cobimetinib) or CXCR4 (motixafortide) was insufficient to achieve meaningful efficacy. Nevertheless, a DCR of 35.7% was observed with third-line atezolizumab plus cobimetinib in a small sample size. Previously, the single-arm phase II COMBAT/KEYNOTE-202 study suggested that the addition of chemotherapy to motixafortide and pembrolizumab in second- or later-line settings could improve the ORR and DCR in patients with PDAC.^33^ It is possible that combining atezolizumab plus motixafortide with chemotherapy might have improved outcomes, as seen in COMBAT/KEYNOTE-202. In the phase Ib JAVELIN study of avelumab plus binimetinib in patients with metastatic PDAC, one PR (ORR 8.3%) occurred along with dose-limiting toxicities,^35^ suggesting challenges to developing CIT combinations with MEK inhibitors. Preclinical studies suggested that combining simlukafusp alfa with an anti-PD-L1 agent strongly enhances the activation of preexisting antigen-specific T cells in the tumor and also that adding a CD40 agonistic antibody could enhance long-lasting anti-tumor efficacy.^20^ Adding another agent to the atezolizumab-simlukafusp alfa combinations evaluated in this study might have improved outcomes. Studies with simlukafusp alfa plus atezolizumab demonstrated clinical activity (ORR 27%) and manageable safety in metastatic cervical cancer in a phase II study.^36^ These studies also showed that the agent was more clinically active in a phase Ib study in renal cell carcinoma when combined with atezolizumab plus bevacizumab than atezolizumab or bevacizumab alone.^37^ The favorable signs of clinical activity we observed support further exploration of simlukafusp alfa in cancer immunotherapy combinations.

There were several limitations to this study, including (1) the small number of observed responses; (2) a lack of germline and somatic sequencing data that precluded evaluating associations between biomarkers and response; (3) the small sample size in each arm dictated by the pre-specified preliminary phase evaluation; and (4) limited treatment combinations that were available as third-line options for patients who progressed from Stage 1 to Stage 2. Although the study was to evaluate first-line and potential new second- and third-line combinations, no additional late-line combinations were introduced before it concluded. Ultimately, only a small fraction of patients (21 of 104 patients who received these second-line regimens [20%]) were eligible for third-line therapy, due in part to the availability of third-line treatment options at the time the study was conducted, highlighting the limited drug development opportunities for late-line treatments for metastatic PDAC and the need for continued research into new treatment options for pancreatic cancer. In addition, the “rolling-arms” study design on which the MORPHEUS platform was based may have the limitation of not being able to adapt to evolving changes in standard of care for the control arm.^28^ However, for pancreatic cancer, this was not the case, and the MORPHEUS design enabled broad comparisons of multiple treatment combinations for PDAC in a short time. The MORPHEUS design resulted in a complex study with multiple treatment arms, various safety assessment requirements, and different timeframes for initiation or conclusion of treatment arms, which introduced operational challenges for some clinical sites. Consequently, the study may have limited enrollment to patients who had access to large academic centers capable of carrying out such a complex study. The inherent limitations of an open-label trial may also have introduced performance or detection biases, including patients in the experimental arms potentially and/or unintentionally receiving care different from patients in the control arm, or patients in the control arm being unwilling to stay in the trial because they were not receiving experimental treatment or were being transitioned quickly off the standard of care to receive alternative therapies.

In conclusion, the MORPHEUS-PDAC platform results showed that combining atezolizumab with motixafortide, cobimetinib, or simlukafusp alfa had limited efficacy as second- or third-line treatment for metastatic PDAC that had progressed following prior chemotherapy. Nevertheless, this umbrella study design permitted not only the evaluation of several new combinations in a relatively short time period but also provided randomized efficacy and safety data for second-line gemcitabine plus *nab*-paclitaxel and mFOLFOX6 regimens in these patients. This innovative study design could serve as a model for a new platform study, such as one that rapidly tests multiple treatment combinations involving KRAS inhibitors, aiming to preemptively address drug resistance mechanisms.

## Supplementary Material

oyag023_Supplementary_Data

## Data Availability

For eligible studies qualified researchers may request access to individual patient-level clinical data through a data request platform. At the time of writing, this request platform is Vivli (https://vivli.org/ourmember/roche/). For up-to-date details on Roche’s Global Policy on the Sharing of Clinical Information and how to request access to related clinical study documents, see: https://go.roche.com/data_sharing. Anonymized records for individual patients across more than one data source external to Roche cannot, and should not, be linked due to a potential increase in risk of patient re-identification.
