# Supplementary material for: Atezolizumab and motixafortide, cobimetinib or simlukafusp alfa in pretreated advanced pancreatic cancer: phase I/IIb MORPHEUS-PDAC umbrella study
Source: Oncologist. 2026 Feb 25;31(4):oyag023. doi: 10.1093/oncolo/oyag023 (PMC12971112; doi:10.1093/oncolo/oyag023)

**Supplementary Data**

**Supplementary Table 1.** Patient disposition.

|  | **Stage 1** | | | | | | **Stage 2** | | |
| --- | --- | --- | --- | --- | --- | --- | --- | --- | --- |
|  | **Atezo + moti** | **Atezo + cobi** | **Atezo + sim q2w** | **Atezo + sim q3w** | **mFOLFOX6** | **Gem + *nab*-P** | **Atezo + cobi** | **Atezo + sim q2w** | **Atezo + sim q3w** |
| **Total randomized, n** | 16 | 15 | 15 | 16 | 25 | 25 | – | – | – |
| **Received ≥1 dose of study treatment, n** | 15 | 14 | 14 | 15 | 23 | 23 | 14 | 1 | 6 |
| **Stage 1 or Stage 2 status, n (%)** |  |  |  |  |  |  |  |  |  |
| Continued into long-term follow-up | 11 (73) | 10 (71) | 14 (93) | 14 (88) | 14 (56) | 13 (52) | 12 (86) | 1 (100) | 6 (100) |
| Continued into Stage 2 | 3 (20) | 0 | 0 | 0 | 6 (24) | 8 (32) | - | - | - |
| Discontinued from Stage 1 | 1 (7) | 4 (29) | 1 (7) | 2 (13) | 5 (20) | 3 (12) | - | - | - |
| Discontinued from Stage 2 | - | - | - | - | - | - | 2 (14) | 1 (100) | 6 (100) |
| **Study status at CCOD, n (%)** |  |  |  |  | 8 (16) |  |  |  |  |
| Ongoing | 0 | 1 (7) | 1 (7) | 0 | - | 1 (2) | 1 (7) | 0 | 0 |
| Discontinued from study | 15 (100) | 13 (93) | 14 (93) | 16 (100) |  | 49 (98) | 13 (93) | 1 (100) | 6 (100) |
| Death | 14 (93) | 10 (71) | 13 (87) | 14 (87) | 1 (2) | 40 (80) | 12 (88) | 1 (100) | 4 (67) |
| Withdrawal by patient | 1 (7) | 3 (21) | 0 | 1 (6) | 49 (98) | 5 (10) | 1 (7) | 0 | 1 (17) |
| Other | 0 | 0 | 1 (7) | 0 | 40 (80) | 3 (6) | 0 | 0 | 0 |
| Loss to follow-up | 0 | 0 | 0 | 0 | 5 (10) | 1 (2) | 0 | 0 | 1 (17) |
| **Reasons for treatment discontinuation** |  |  |  |  |  |  |  |  |  |
| **n** | **15** | **14** | **14** | **15** | **23** | **23** | **14** | **1** | **6** |
| PD, n (%) | 13 (87) | 7 (50) | 14 (100) | 10 (67) | 17 (74) | 12 (74) | 5 (36) | 1 (100) | 4 (67) |
| Withdrawal by patient, n (%) | 1 (7) | 4 (29) | 0 | 2 (13) | 4 (17) | 2(9) | 2 (14) | 0 | 0 |
| Death, n (%) | 0 | 1 (7) | 0 | 1 (7) | 0 | 1 (4) | 1 (7) | 0 | 0 |
| Adverse event, n (%) | 0 | 0 | 0 | 0 | 1 (4) | 0 | 1 (7) | 0 | 0 |
| Physician decision, n (%) | 1 (7) | 2 (14) | 0 | 1 (7) | 1 (4) | 1 (4) | 4 (29) | 0 | 1 (17) |
| Pregnancy, n (%) | 0 | 0 | 0 | 0 | 0 | 1 (4) | 0 | 0 | 0 |
| Symptomatic deterioration, n (%) | 0 | 0 | 0 | 1 (7) | 0 | 1(4) | 1 (7) | 0 | 1 (17) |

**Supplementary Table 2**. Baseline laboratory characteristics with potential prognostic value

|  | **Stage 1 (second-line treatment)** | | | | | **Stage 2 (third-line treatment)** | | | |
| --- | --- | --- | --- | --- | --- | --- | --- | --- | --- |
|  | **Atezo + moti (n=15)** | **Atezo + cobi (n=14)** | **Atezo + sim q2w (n=15)** | **Atezo + sim q3w (n=16)** | **mFOLFOX6**  **(n=25)** | **Gem + *nab*-P (n=25)** | **Atezo + cobi (n=14)** | **Atezo+ sim q2w  (n=1)** | **Atezo + sim q3w (n=6)** |
| **Baseline albumin, n (%)** | | | | | | | | | |
| ≥3.5 g/dL | 13 (86.7) | 9 (64.3) | 9 (64.3) | 14 (87.5) | 16 (64.0) | 21 (84.0) | 11 (78.6) | 1 (100) | 2 (33.3) |
| **Baseline CRP, n (%)** | | | | | | | | | |
| ≤1.2 mg/dL | 13 (86.7) | 8 (57.1) | 8 (57.1) | 14 (87.5) | 14 (56.0) | 20 (80.0) | 9 (64.3) | 0 | 3 (60.0) |
| **Baseline LDH, n (%)** | | | | | | | | | |
| <1.5 × ULN | 14 (93.3) | 13 (92.9) | 14 (100) | 15 (93.8) | 23 (92.0) | 24 (96.0) | 11 (78.6) | 0 | 6 (100) |
| 1.5 × ULN to <2.5 × ULN | 1 (6.7) | 1 (7.1) | 0 | 1 (6.3) | 1 (4.0) | 1 (4.0) | 2 (14.3) | 0 | 0 |
| ≥2.5 × ULN | 0 | 0 | 0 | 0 | 1 (4.0) | 0 | 1 (7.1) | 1 (100) | 0 |
| **Baseline NLR, n (%)** | | | | | | | | | |
| <5 | 5 (83.3) | 5 (62.5) | 10 (71.4) | 13 (81.3) | 15 (60.0) | 19 (76.0) | 6 (54.5) | 0 | 3 (50.0) |

Abbreviations: Atezo, atezolizumab; cobi, cobimetinib; CRP, C-reactive protein; FU, fluorouracil; gem, gemcitabine; LDH, lactate dehydrogenase; mFOLFOX-6, 5-fluorouracil, leucovorin, and oxaliplatin; *nab*-P, *nab*-paclitaxel; NLR, neutrophil-to-lymphocyte ratio; q2w, every 2 weeks; q3w, every 3 weeks; sim, simlukafusp alfa; ULN, upper limit of normal

**Supplementary Table 3.** Treatment-related AEs reported by ≥20% of patients in any combination treatment arm

| n (%) | **Stage 1 (second-line treatment)** | | | | | **Stage 2 (third-line treatment)** | | |
| --- | --- | --- | --- | --- | --- | --- | --- | --- |
|  | **Atezo + moti (n=15)** | **Atezo + cobi (n=14)** | **Atezo + sim q2w (n=14)** | **Atezo + sim q3w (n=15)** | **Gem + *nab*-P or mFOLFOX6 (n=46)** | **Atezo + cobi (n=14)** | **Atezo + sim q2w (n=1)** | **Atezo + sim q3w (n=6)** |
| Fatigue | 8 (53.3) | 4 (28.6) | 6 (42.9) | 3 (20) | 10 (21.7) | 1 (7.1) | 0 | 1 (16.7) |
| Pruritus | 3 (20) | 1 (7.1) | 3 (21.4) | 0 | 2 (13.3) | 0 | 0 | 2 (33.3) |
| Nausea | 3 (20 | 3 (21.4) | 8 (57.1) | 2 (13.3) | 18 (39.1) | 2 (14.3) | 0 | 2 (33.3) |
| Vomiting | 1 (6.7) | 3 (21.4) | 4 (28.6) | 3 (20) | 11 (23.9) | 3 (21.4) | 0 | 1 (16.7) |
| Diarrhea | 0 | 5 (35.7) | 1 (7.1) | 0 | 4 (8.7) | 6 (42.9) | 0 | 1 (16.7) |
| Pyrexia | 0 | 3 (21.4) | 9 (64.3) | 8 (53.3) | 6 (13) | 0 | 0 | 3 (50) |
| Chills | 0 | 1 (7.1) | 6 (42.9) | 9 (60) | 1 (2.2) | 1 (7.1) | 0 | 2 (33.3) |
| Rash | 1 (6.7) | 0 | 2 (14.3) | 1 (6.7) | 2 (4.3) | 3 (21.4) | 0 | 0 |
| Asthenia | 1 (6.7) | 0 | 1 (7.1) | 0 | 3 (6.5) | 1 (7.1) | 0 | 0 |
| Dizziness | 0 | 0 | 4 (28.6) | 2 (13.3) | 0 | 2 (14.3) | 0 | 0 |
| Aspartate aminotransferase increased | 0 | 3 (21.4) | 7 (50.0) | 6 (40) | 6 (13) | 0 | 0 | 1 (16.7) |
| Alanine aminotransferase increased | 0 | 1 (7.1) | 5 (35.7) | 5 (33.3) | 5 (10.9) | 0 | 0 | 1 (16.7) |
| Blood alkaline phosphatase increased | 0 | 1 (7.1) | 4 (28.6) | 2 (13.3) | 2 (4.3) | 1 (7.1) | 0 | 1 (16.7) |
| Platelet count decreased | 0 | 0 | 1 (7.1) | 3 (20) | 7 (15.2) | 0 | 0 | 0 |
| Blood bilirubin increased | 0 | 0 | 4 (28.6) | 2 (13.3) | 0 | 0 | 0 | 0 |
| Hypotension | 0 | 0 | 0 | 5 (33.3) | 0 | 0 | 0 | 0 |
| Injection-site reaction | 11 (73.3) | 0 | 0 | 0 | 0 | 0 | 0 | 0 |

Abbreviations: Atezo, atezolizumab; cobi, cobimetinib; gem, gemcitabine; *nab*-P, *nab*-paclitaxel; sim, simlukafusp alfa.

**Supplementary Figure 1.** MORPHEUS-PDAC study design.

Abbreviations: 2L, second line; 3L, third line; chemo, chemotherapy; FU, fluorouracil; mFOLFOX-6, 5-fluorouracil, leucovorin, and oxaliplatin; PDAC, pancreatic ductal adenocarcinoma; q2w, every 2 weeks; q3w, every 3 weeks; R, randomization.


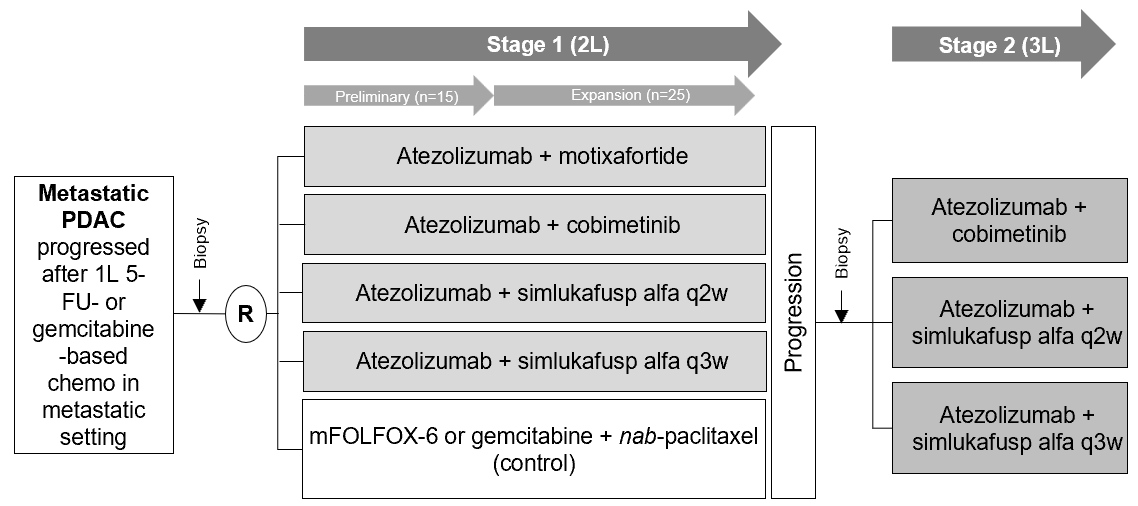


**Supplementary Figure 2.** Clinical activity with second-line treatment combinations in Stage 1. **A,** Swimlane plots. **B,** Spaghetti plots.

Abbreviations: 2L, second line; 3L, third line; mFOLFOX-6, 5-fluorouracil, leucovorin, and oxaliplatin; NA, not available; NE, not evaluable; PD, disease progression; PR, partial response; q2w, every 2 weeks; q3w, every 3 weeks; SD, stable disease; tx, treatment.

**A**


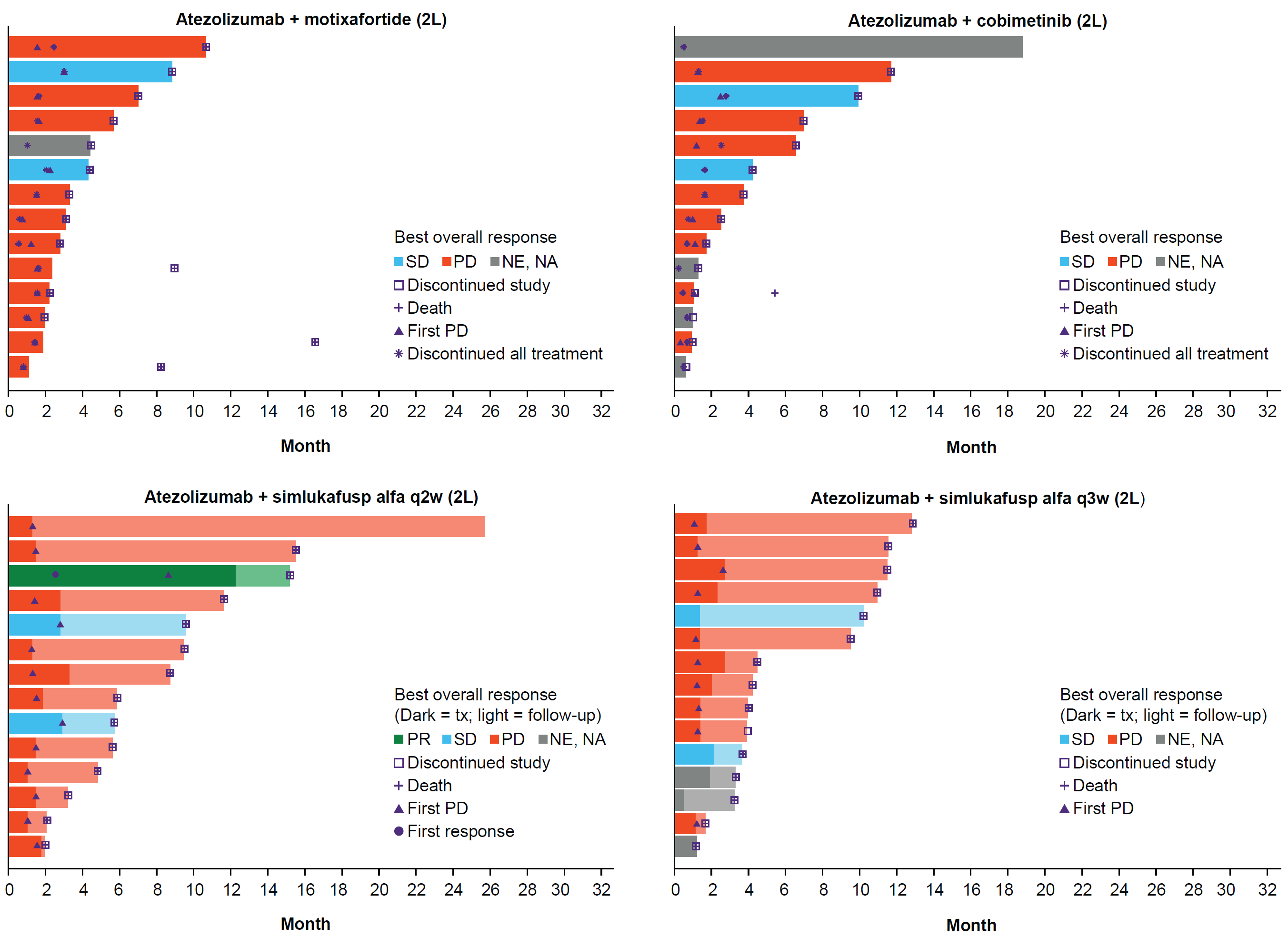


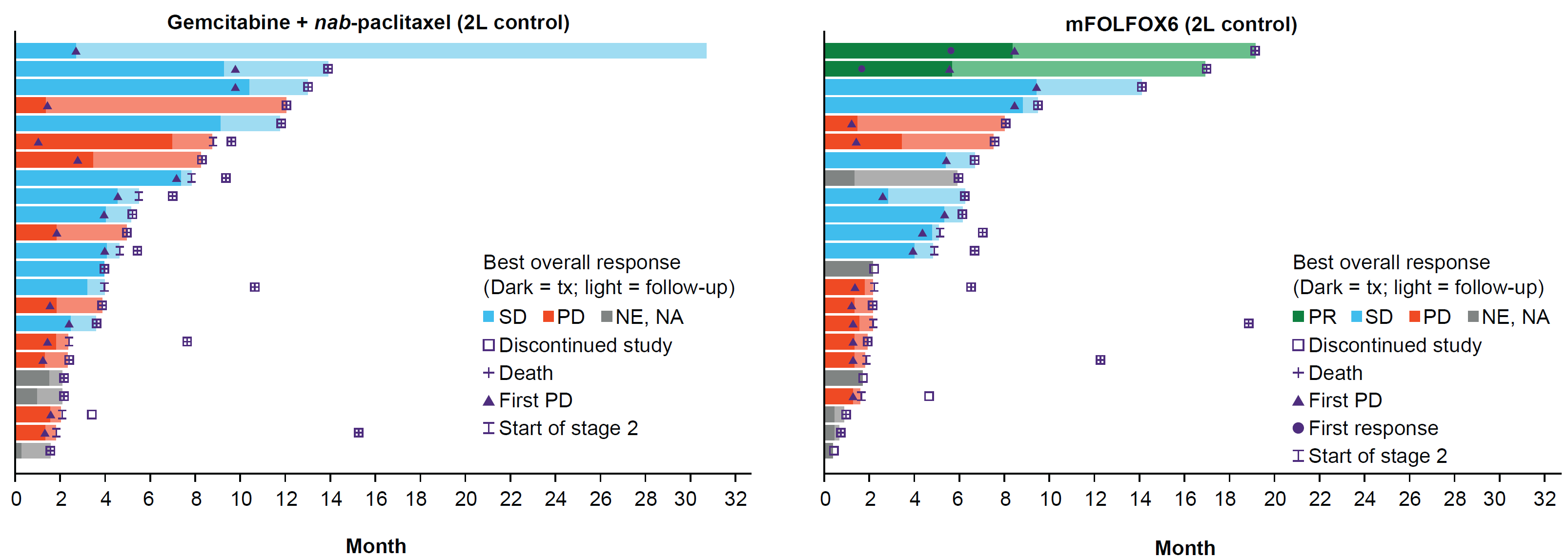


**B**

**
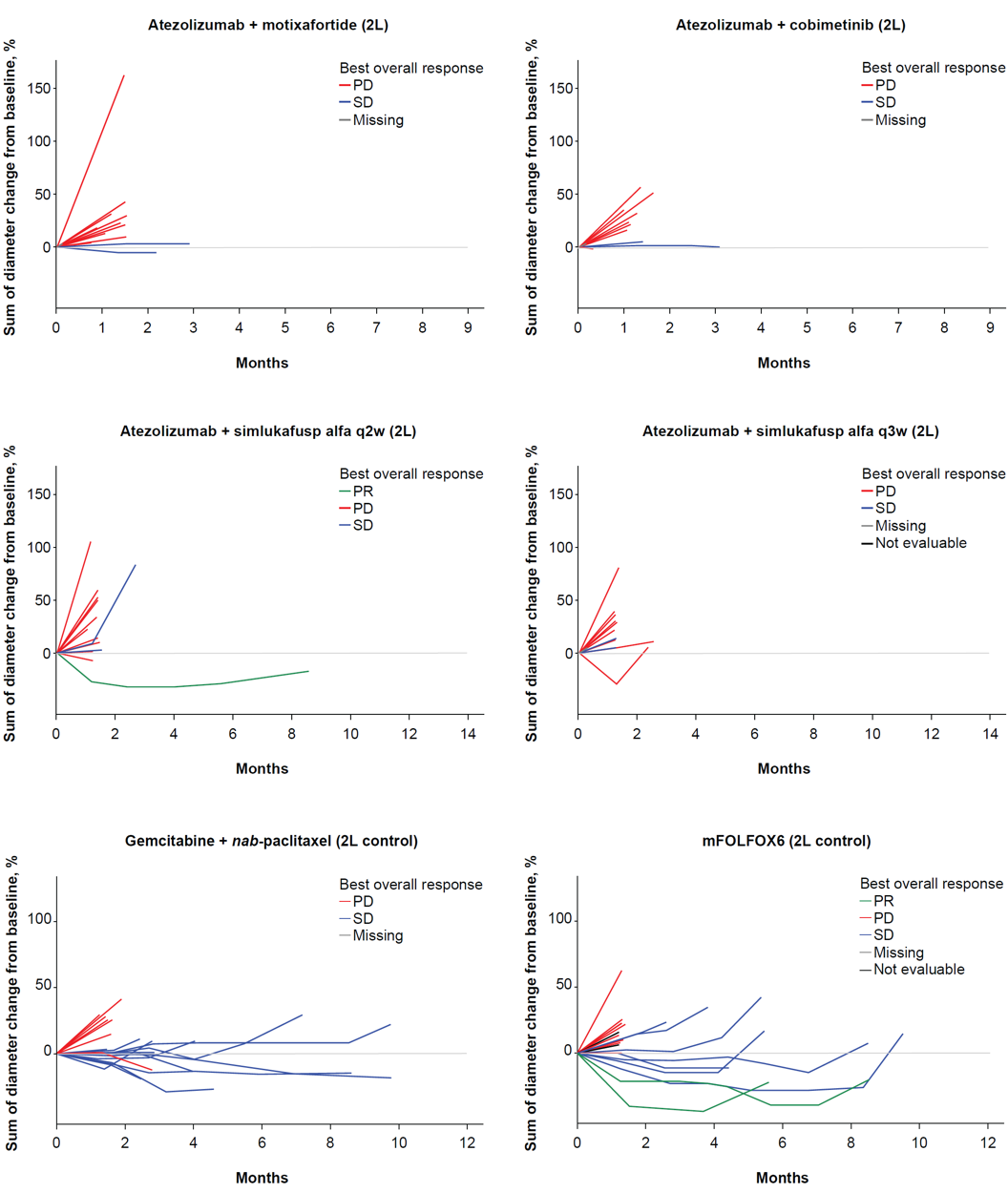
**

**Supplementary Figure 3.** PFS with 2L treatment in Stage 1.

Abbreviations: 2L, second line; Atezo, atezolizumab; CI, confidence interval; cobi, cobimetinib; HR, hazard ratio; mFOLFOX-6, 5-fluorouracil, leucovorin, and oxaliplatin; NE, not evaluable; PFS, progression-free survival; q2w, every 2 weeks; q3w, every 3 weeks.

**Atezolizumab + motixafortide (2L)**

**
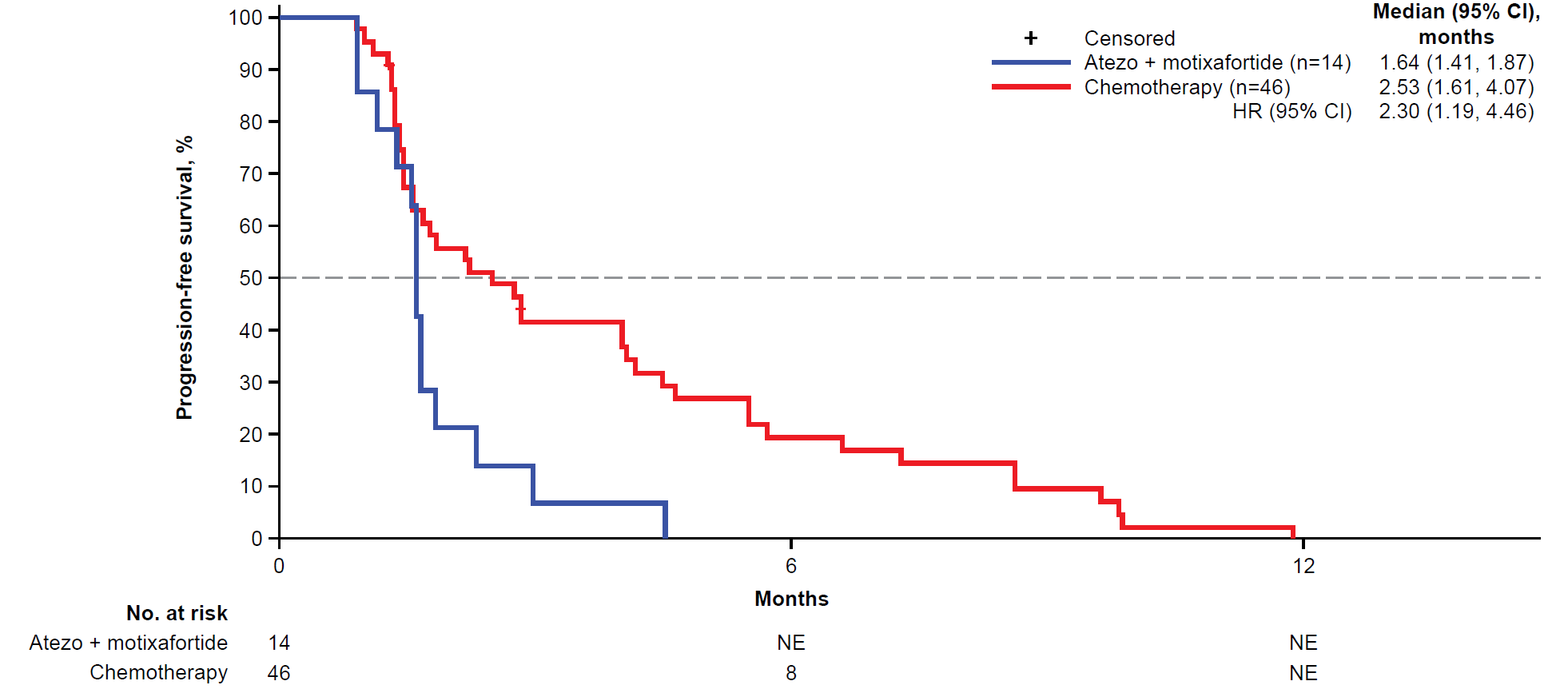
**

**Atezolizumab + cobimetinib (2L)**

**
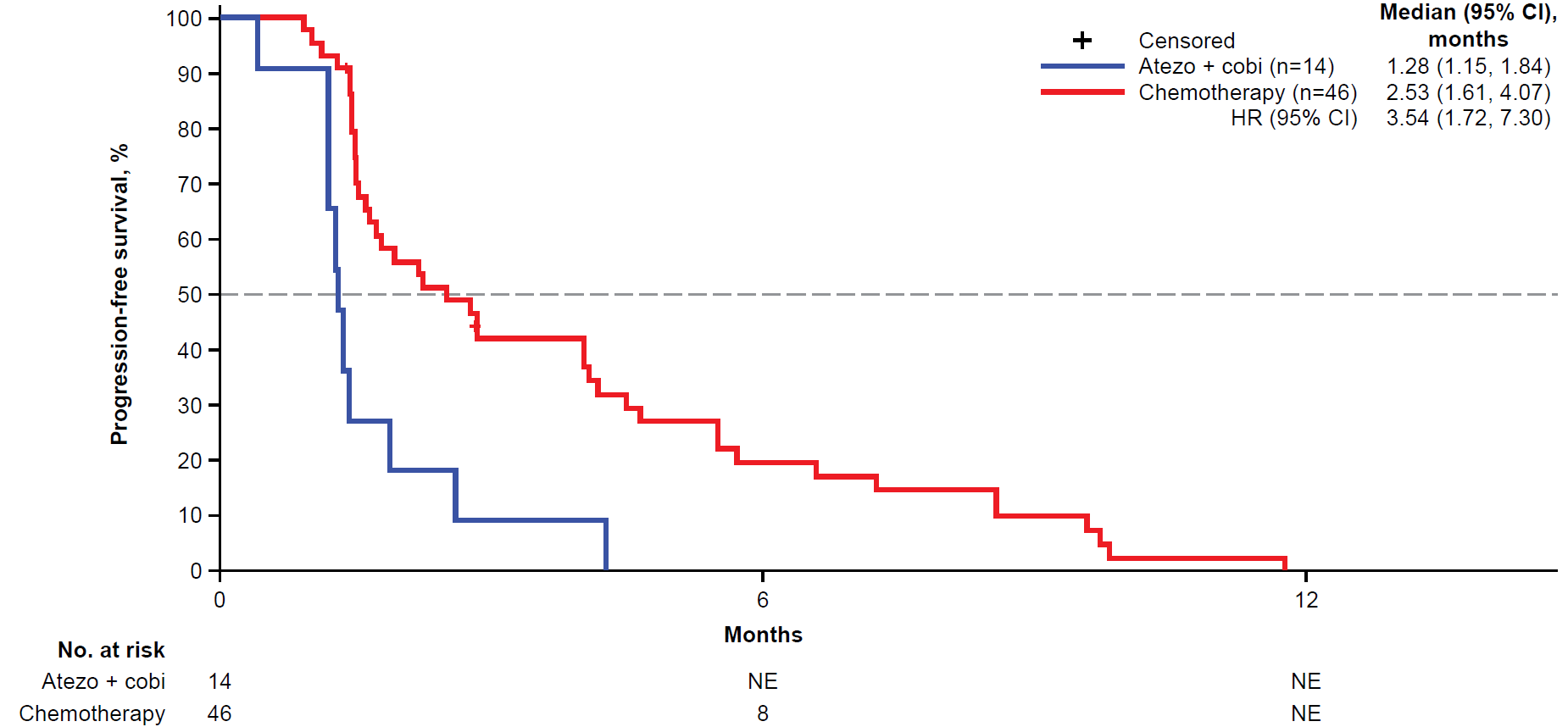
**

**Atezolizumab + simlukafusp alfa q2w (2L)**

**
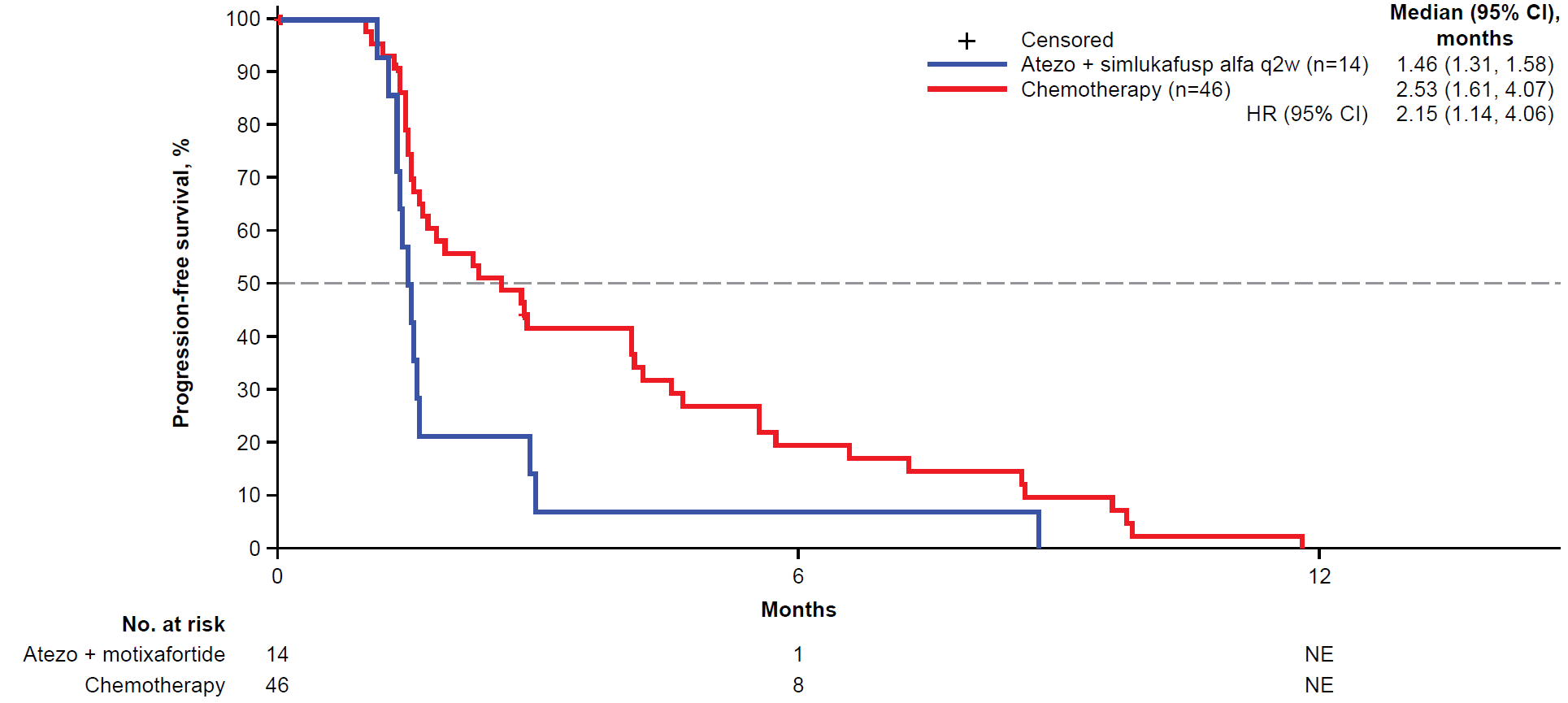
**

**Atezolizumab + simlukafusp alfa q3w (2L)**

**
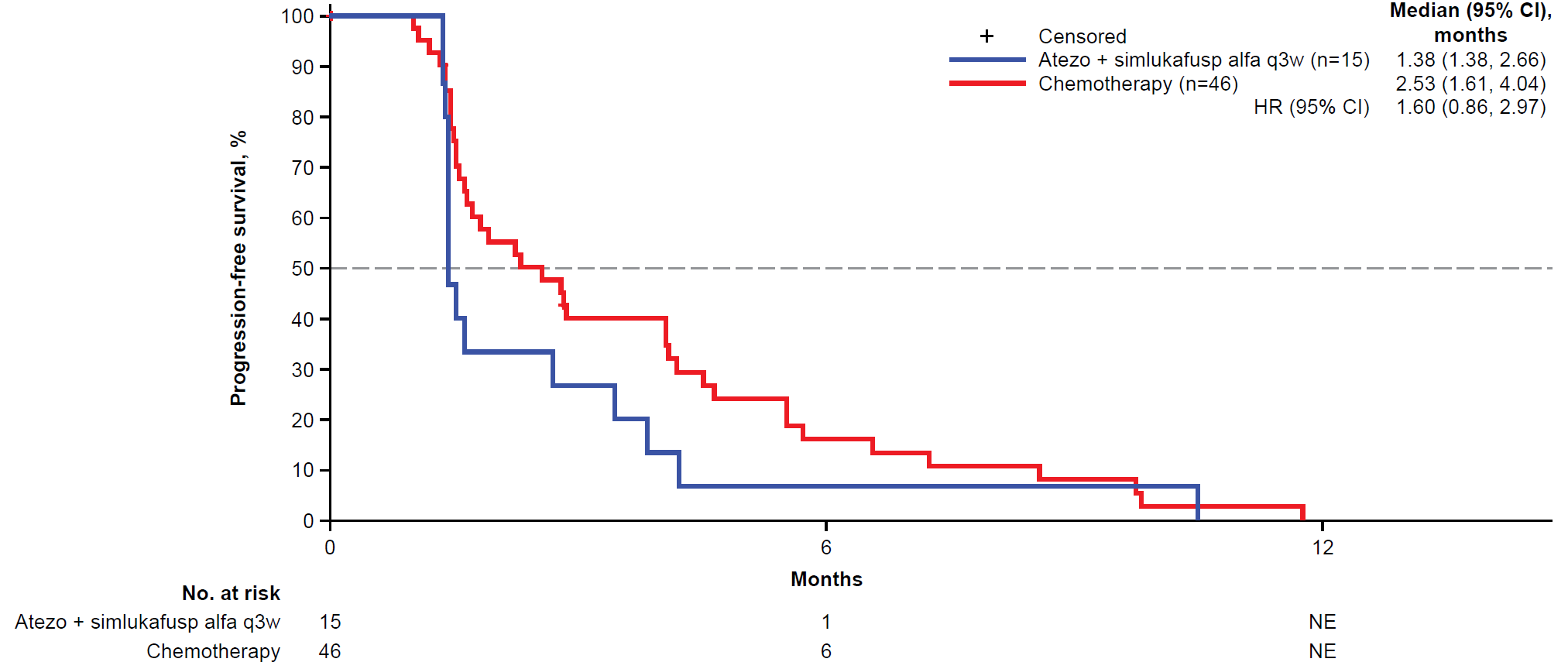
**

**Chemotherapy (2L control)**

**
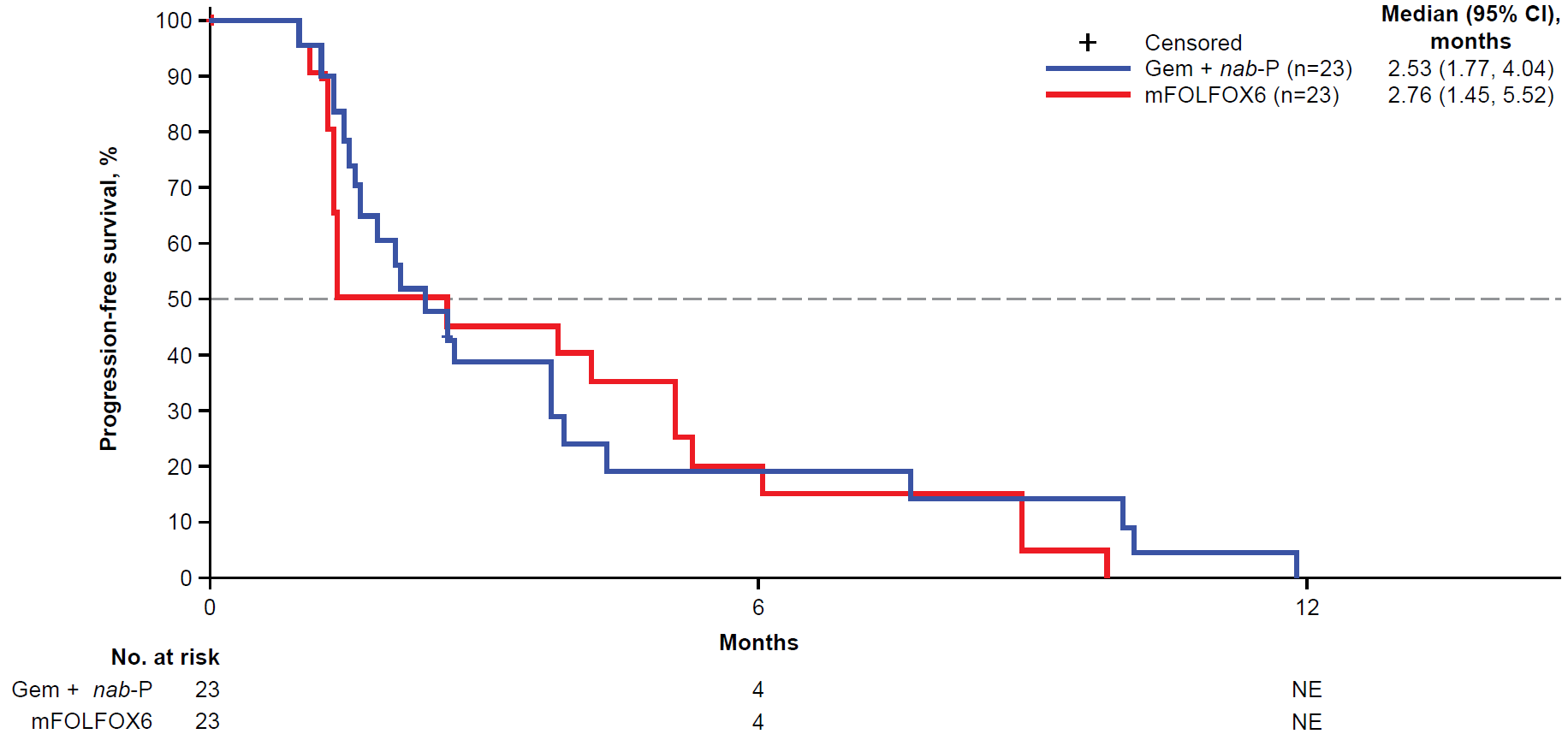
**

**Supplementary Figure 4.** OS with 2L treatment in Stage 1.

Abbreviations: 2L, second line; Atezo, atezolizumab; CI, confidence interval; cobi, cobimetinib; HR, hazard ratio; mFOLFOX-6, 5-fluorouracil, leucovorin, and oxaliplatin; NE, not evaluable; OS, overall survival; q2w, every 2 weeks; q3w, every 3 weeks.

**Atezolizumab + motixafortide (2L)**


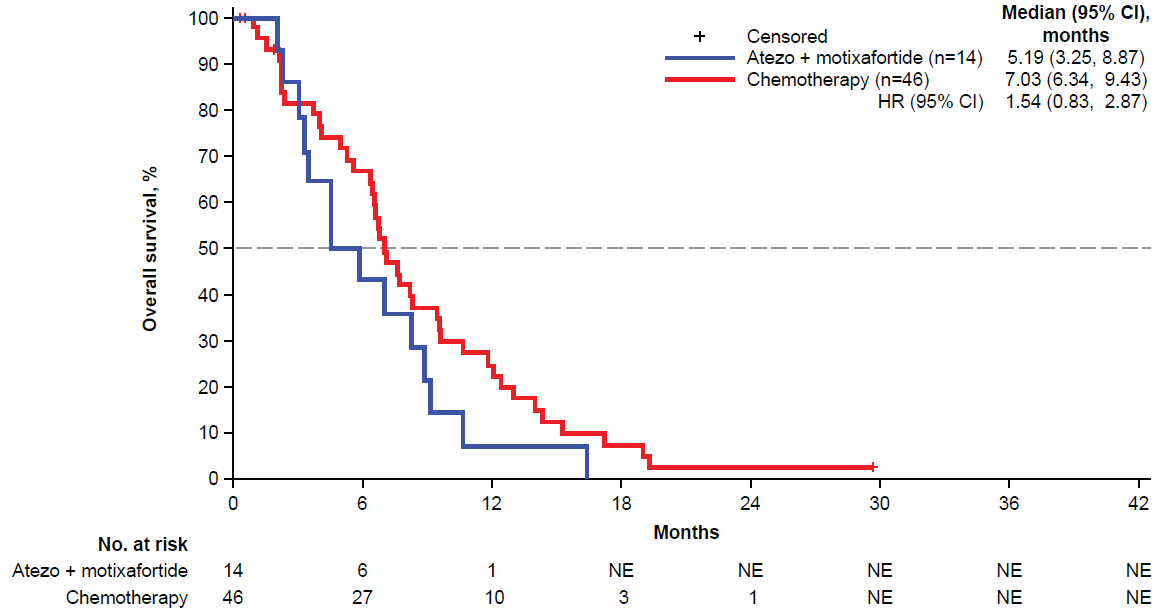


**Atezolizumab + cobimetinib (2L)**


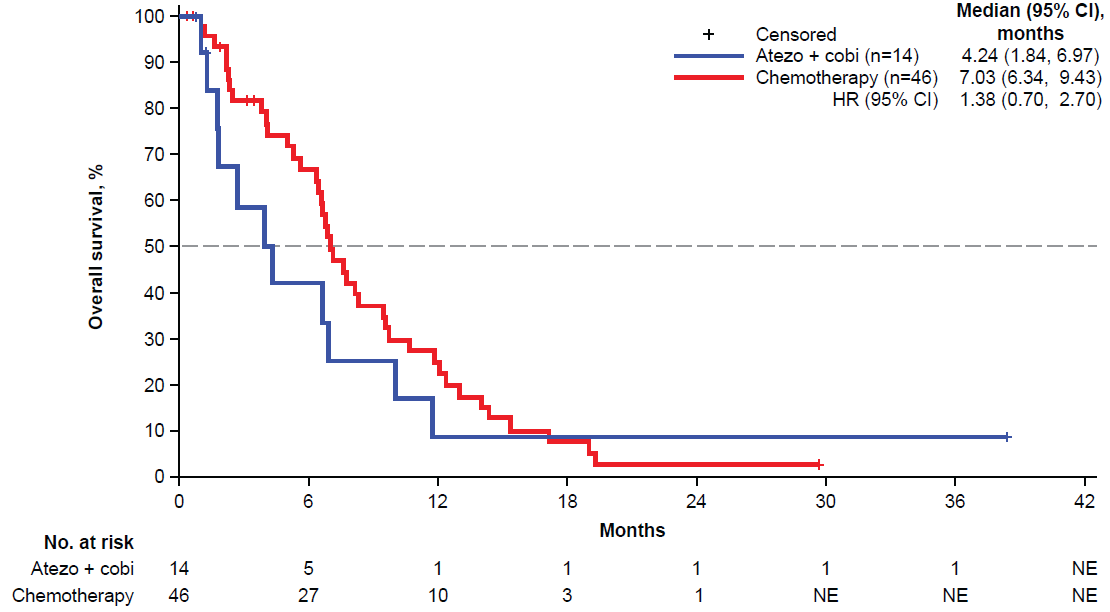


**Atezolizumab + simlukafusp alfa q2w (2L)**


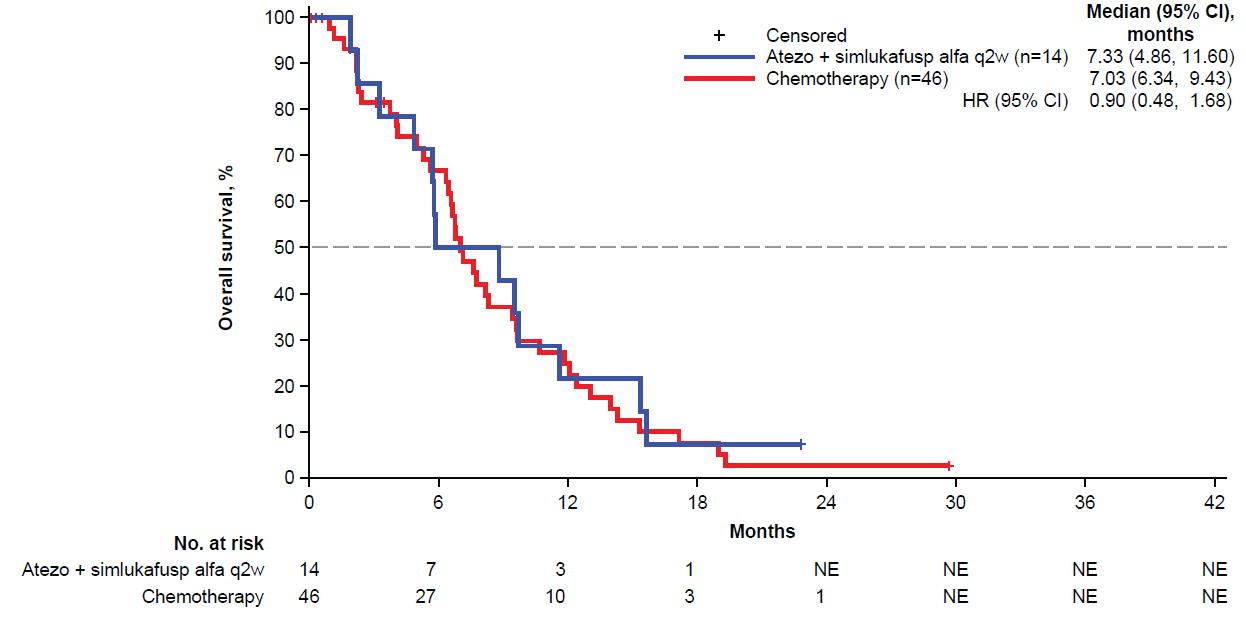


**Atezolizumab + simlukafusp alfa q3w (2L)**


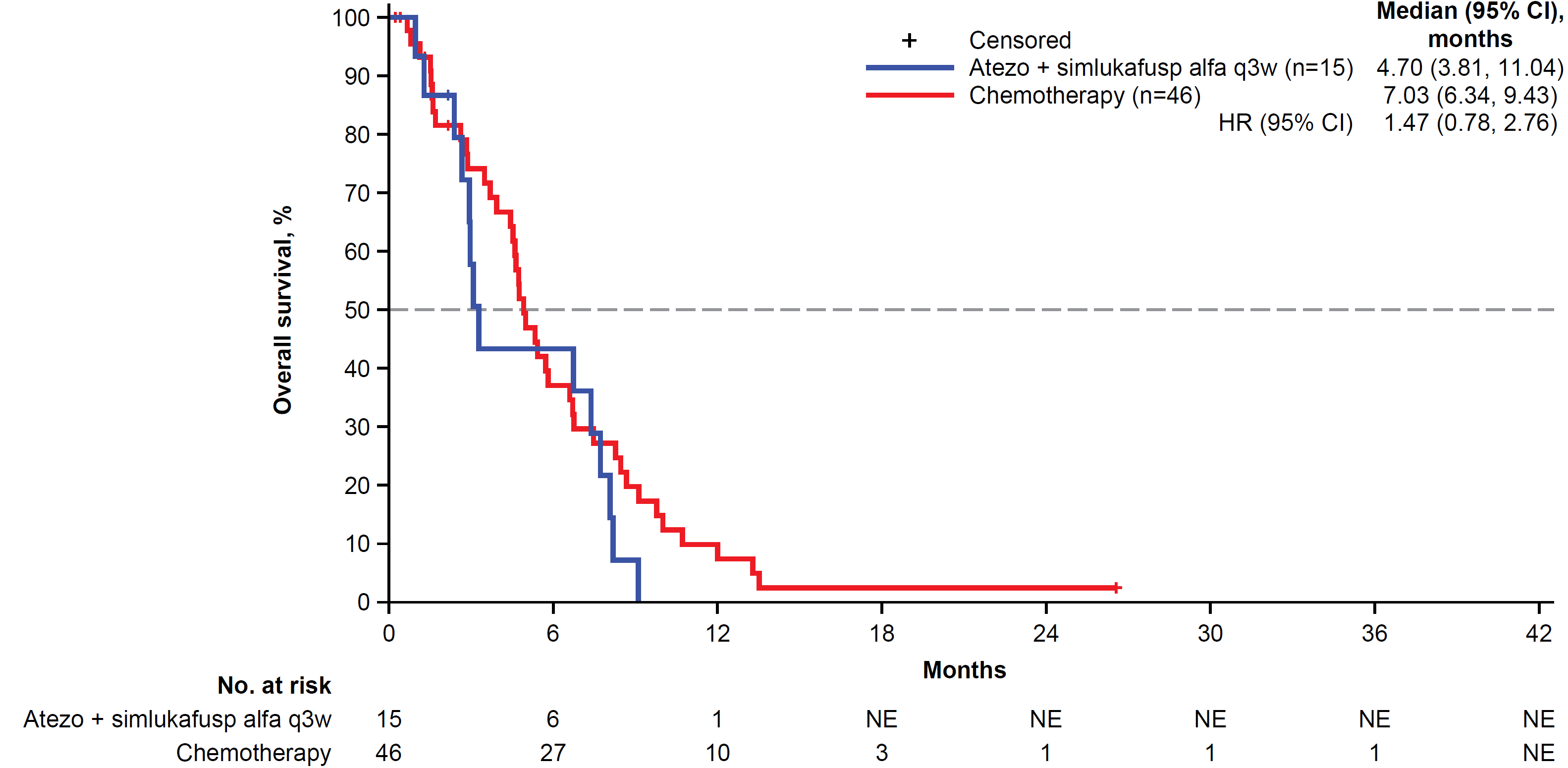


**Chemotherapy (2L control)**

**
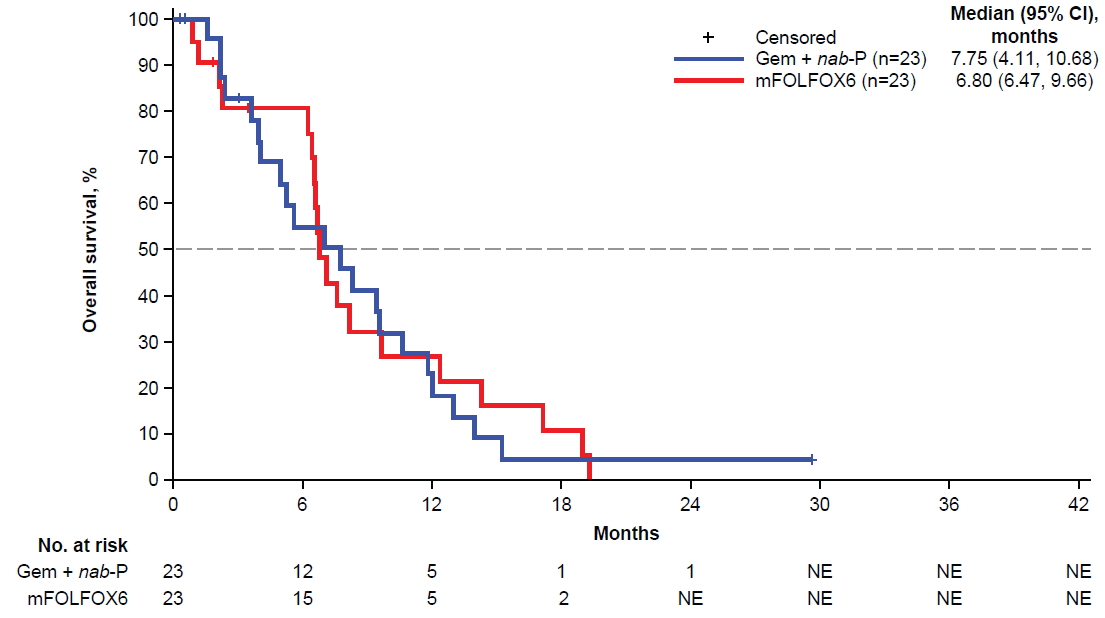
**

**Supplementary Figure 5.** Clinical activity with third-line treatment combinations in Stage 2. **A**, Waterfall plots. **B**, Swimlane plots. **C,** Spaghetti plots.

Abbreviations: 3L, third line; NA, not available; NE, not evaluable; PD, disease progression; PR, partial response; q3w, every 3 weeks; SD, stable disease.

**A**

**
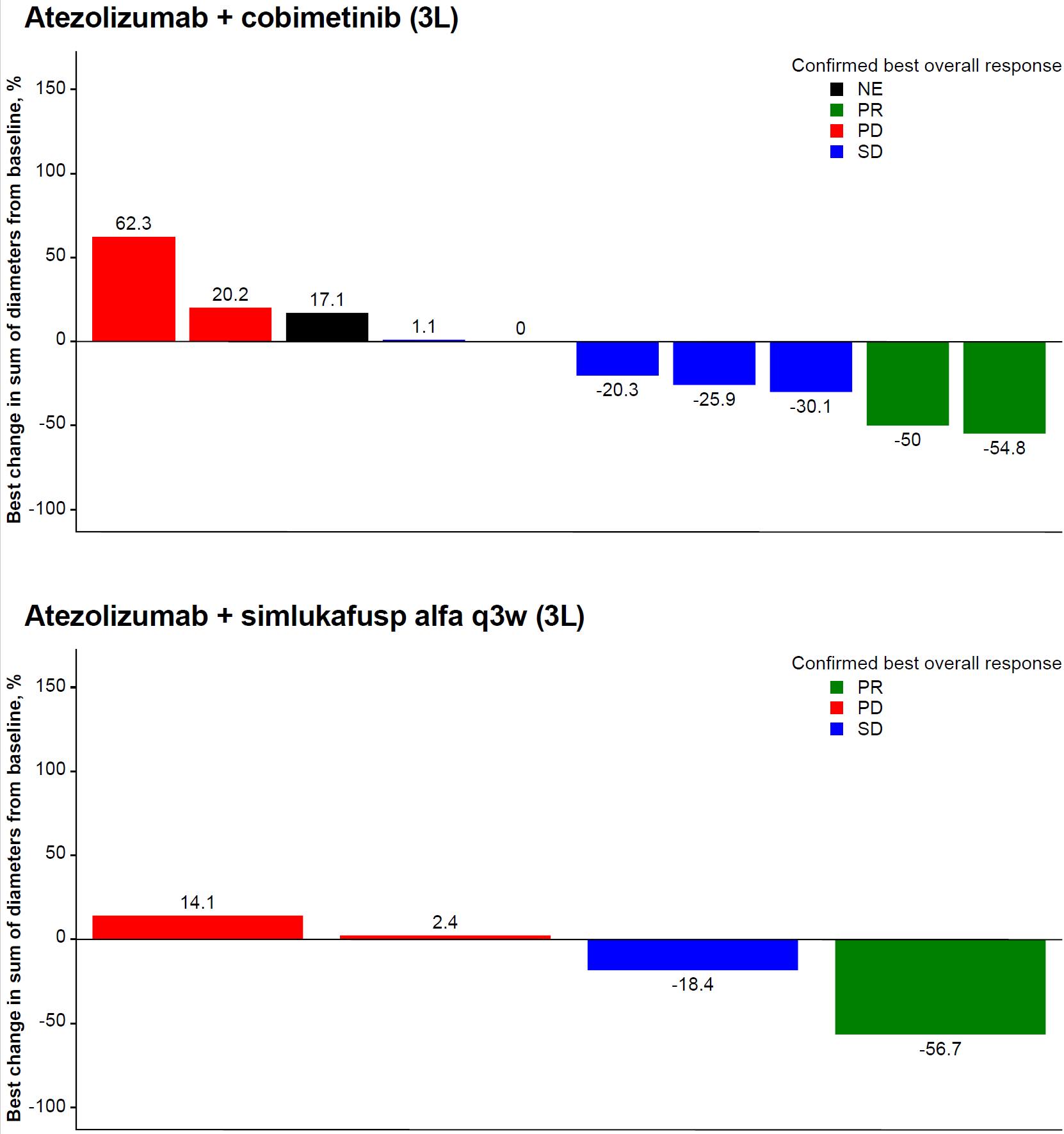
**

**B**

**
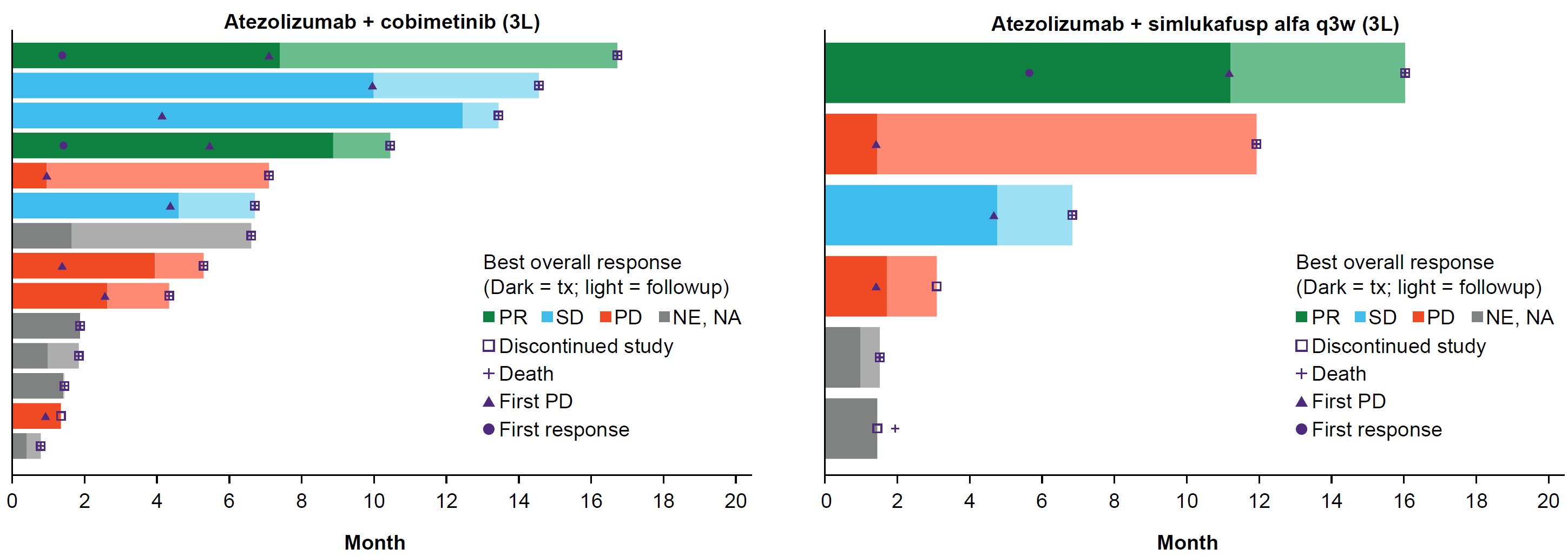
**

**C**

**
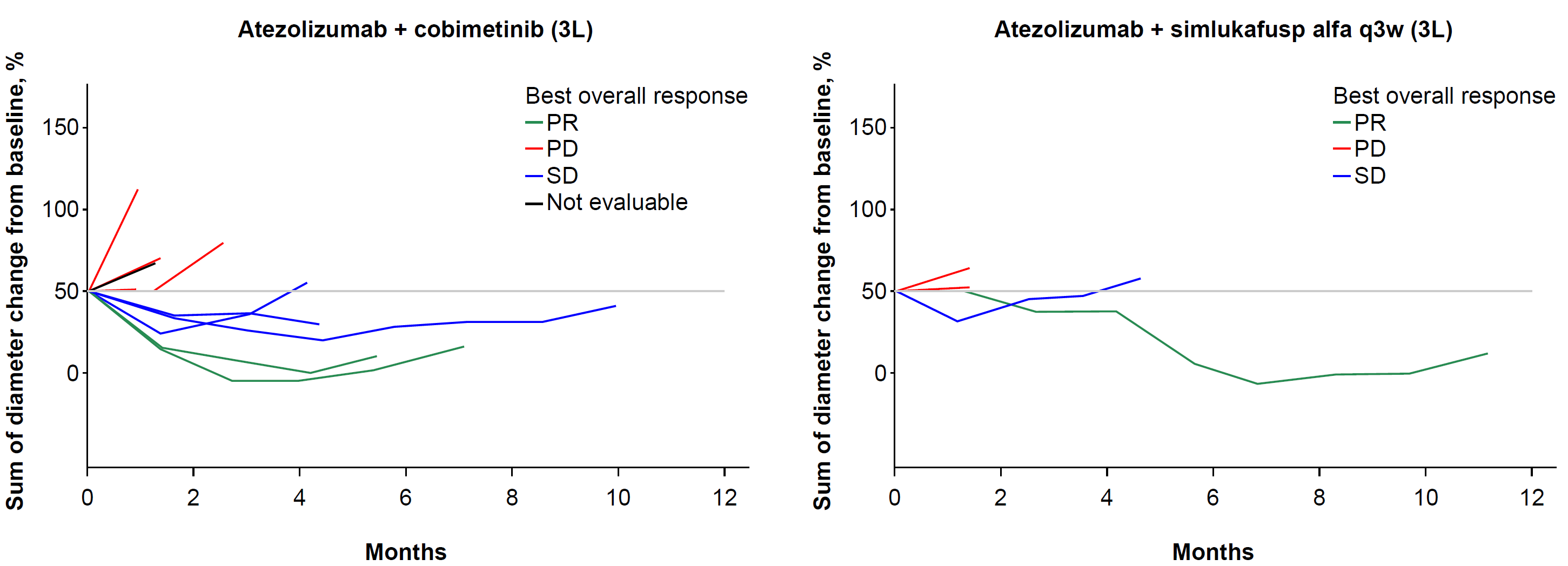
**

**Supplementary Figure 6. (A)** PFS and **(B)** OS with 3L treatment in stage 2.

Abbreviations: 3L, third line; Atezo, atezolizumab; CI, confidence interval; cobi, cobimetinib; NE, not evaluable; OS, overall survival; PFS, progression-free survival; q3w, every 3 weeks.

**A Atezolizumab + cobimetinib (3L)**

**
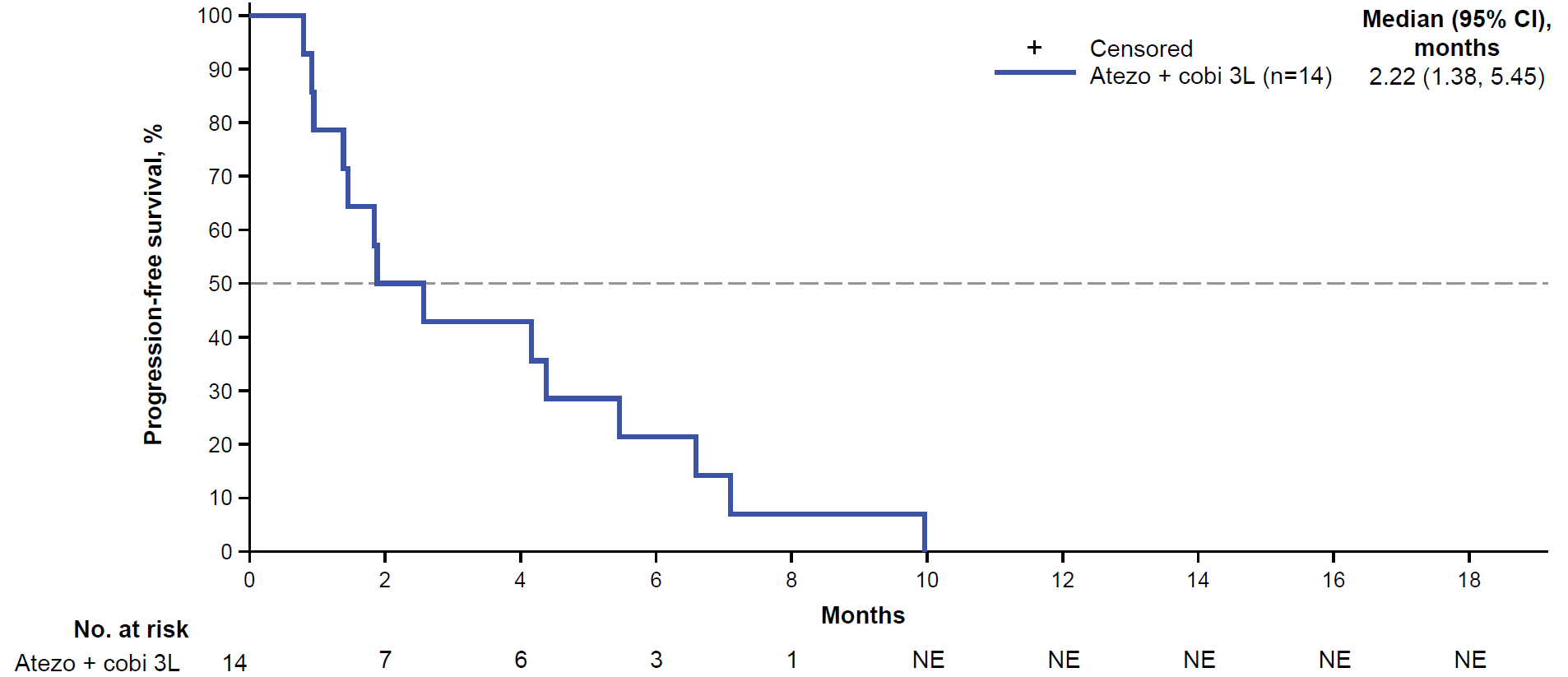
**

**Atezolizumab + simlukafusp alfa q3w (3L)**


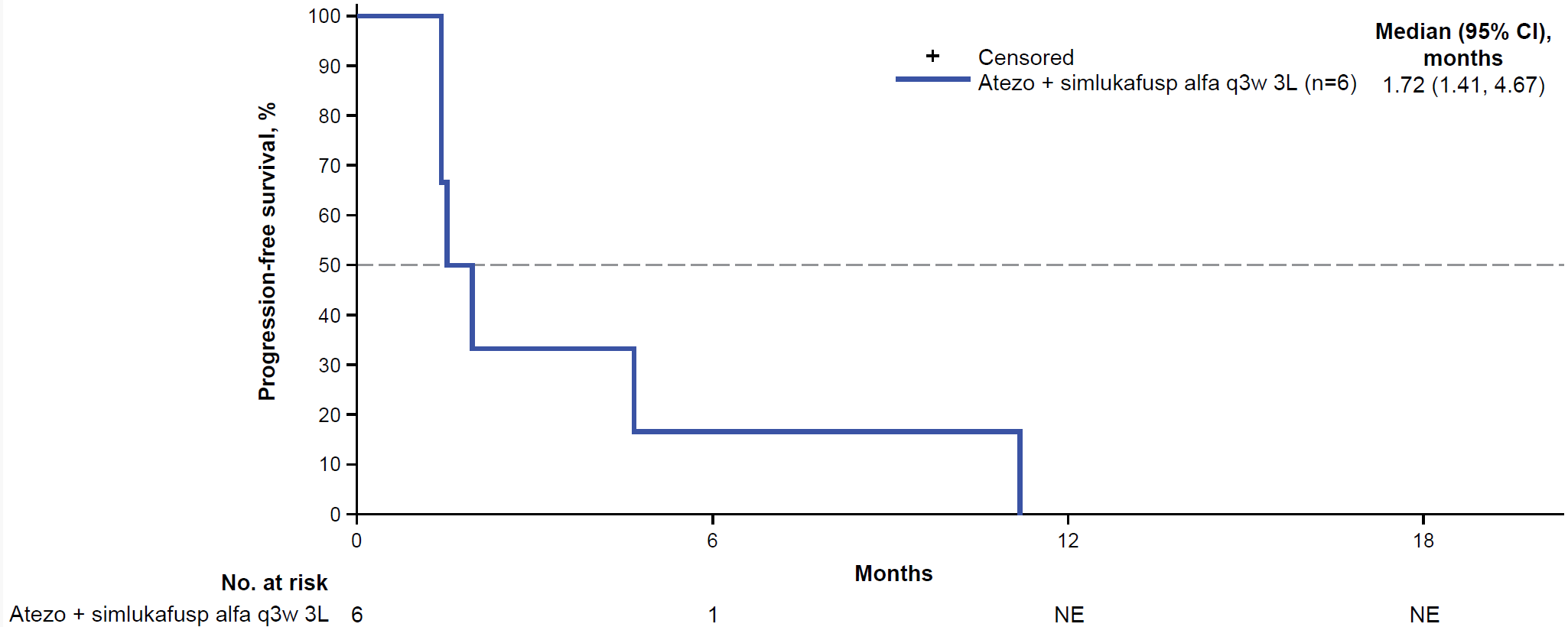


**B Atezolizumab + cobimetinib (3L)**

**
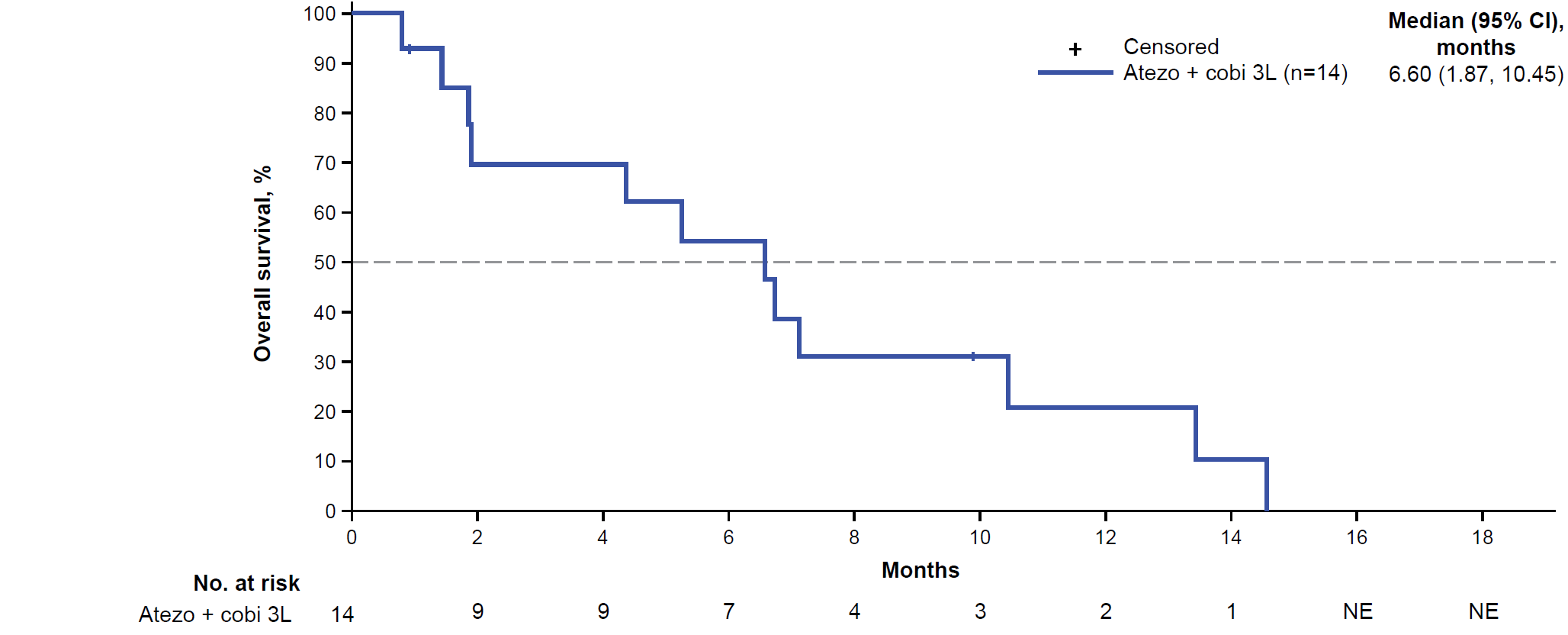
**

**Atezolizumab + simlukafusp alfa q3w (3L)**


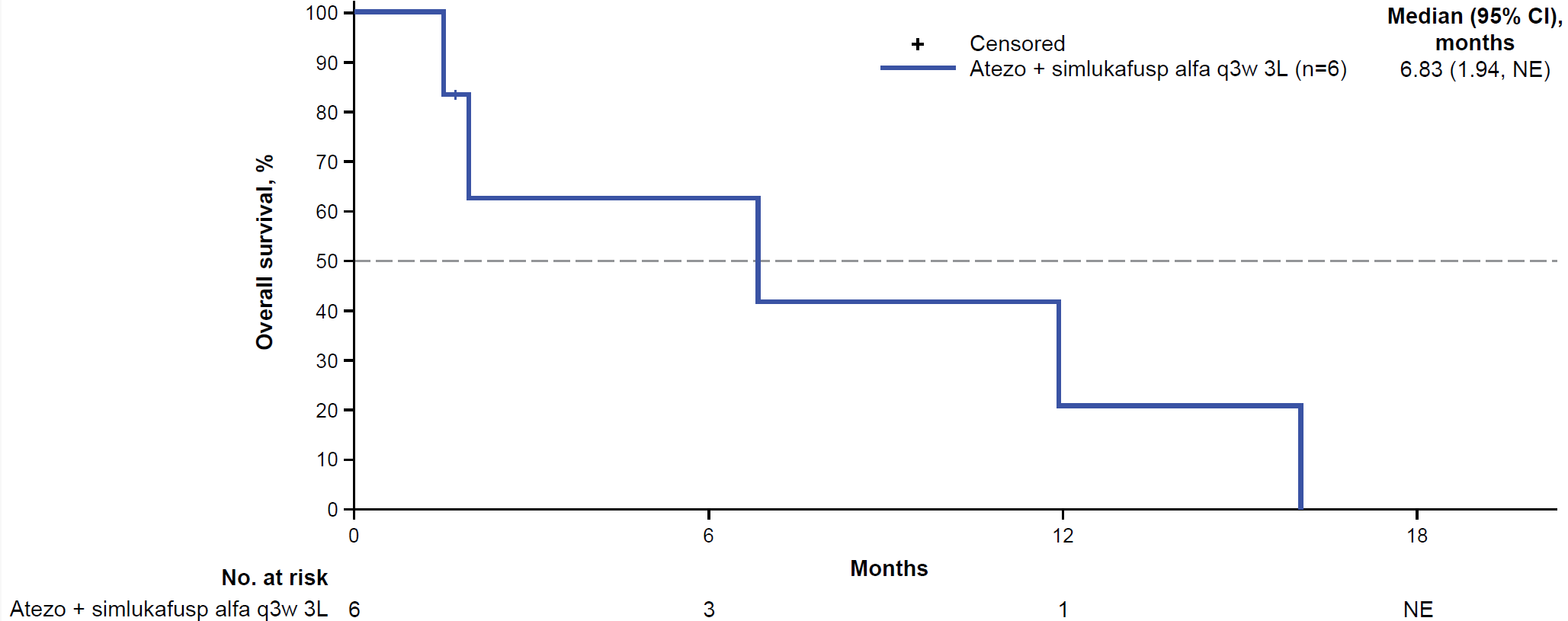

Supplement: oyag023_Supplementary_Data [file oyag023_supplementary_data.docx]
